# “Their Bodies Were Made to Move and Wriggle Right from the Word Go”: A Qualitative Exploration of Family Engagement with Fundamental Movement Skills in Early Childhood

**DOI:** 10.3390/children13040563

**Published:** 2026-04-18

**Authors:** Robert J. Flynn, Andy Pringle, Clare M. P. Roscoe

**Affiliations:** Clinical Exercise Rehabilitation Research Centre, School of Sport and Exercise Science, University of Derby, Derby DE22 1GB, UK; a.pringle@derby.ac.uk

**Keywords:** fundamental movement skills, physical activity, children, caregivers, family, perspectives, determinants

## Abstract

**Highlights:**

**What are the main findings?**

- Both educators and caregivers were unaware of the Chief Medical Officers’ physical activity guidelines for children in the United Kingdom (CMO Guidelines), indicating that current communication channels are ineffective and are leaving families uninformed about key messages on physical activity and fundamental movement skill development.
- Despite parental concerns about their physical capability, grandparents were found to be time-rich, highly motivated, and adaptable in supporting children’s activities, including through indirect means when physically unable to participate.

**What are the implications of the main finding?**

- The CMO Guidelines must be made more visible and accessible through professional training and active promotion across early education, healthcare, and community settings to ensure families receive guidance about the importance of supporting children’s physical activity and fundamental movement skill development.
- Future iterations of the CMO Guidelines should include multi-component advice on physical activity, screen time, and sleep, and should be tailored to and made available in multiple formats, to help grandparents with physical limitations support and engage children in developing their fundamental movement skills.

**Abstract:**

**Background:** Fundamental movement skills (FMS) underpin lifelong physical activity (PA) and health, yet many children are failing to meet age-appropriate standards. Caregivers hold a critical influence over children’s motor development, but little is known about what helps or hinders family participation, including messaging. This study explored the determinants of family FMS engagement in the United Kingdom (UK) during early childhood, addressing unexplored gaps in how guidance reaches families and the role of grandparents in supporting children’s motor development. **Methods:** Twenty-three semi-structured interviews were conducted with 15 caregivers and 8 educators, including 4 grandparents and 2 family hub practitioners who offered original insights. Eleven children aged 3–5 years completed a flexible draw-and-tell task, enabling inclusion of rarely represented 3-year-olds. Thematic analysis was deployed. **Results:** Families and outdoor spaces were pivotal to children’s movement opportunities. However, awareness and understanding of FMS and UK PA guidance were poor, even among educators, disrupting dissemination of information to families. Greater emphasis on PA and FMS concepts within professional development, alongside clearer signposting to resources, more visible public-facing campaigns, and digital formats, could improve how families receive these messages. Tensions emerged between parents’ concerns about grandparents’ physical capability and grandparents’ belief that they could adapt to support children’s development. Unexpectedly, no children drew technology despite screen time frequently displacing active play, hinting at its normalisation and regulatory role in children’s lives. **Conclusions:** To enhance family understanding, value, and participation in FMS, UK policy must evolve to become more visible, relatable, and responsive to diverse family needs.

## 1. Introduction

Fundamental movement skills (FMS) are foundational motor abilities in children that act as the building blocks for more complex movement patterns [1,2]; they are typically subdivided into locomotor skills, object control skills, and balance [3]. Locomotor skills (e.g., running and jumping) allow children to traverse and explore their surroundings [4]. Object control skills (e.g., throwing and catching) involve the coordinated manipulation of external objects [5]. Meanwhile, balance enables children to meet static and dynamic challenges like standing on one foot or travelling along a balance beam [6]. Early and appropriate development of these rudimentary abilities in early childhood (3–5 years) contributes to a child’s physical literacy and lifelong engagement in physical activity (PA) [7], which may lead to a range of physical, psychosocial, and educational benefits [8]. To reach full motor competence, children require suitable environments, opportunities to practice, and appropriate instruction [9]. Therefore, adequate support for FMS in early childhood may establish a base for meaningful PA participation and long-term health and wellbeing.

High-quality professionally delivered programmes within educational settings are considered to be an effective approach of improving children’s FMS [10,11], but these gains are not always maintained over time [11]. As a result, many children still fail to reach expected levels of competence [2,12], and standards may even be in decline in many regions [13,14,15]. This evidence demonstrates the need for alternative or complementary strategies, including engagement with caregivers, alongside acknowledgement that teachers’ expertise in delivering physical education (PE) may vary. Children are strongly influenced by close, reciprocal relationships with their caregivers, who provide emotional security and critical learning opportunities [16]. Given this profound relational bond, caregivers represent a valuable, underused source of support for children’s FMS. Although caregivers tend to have poor awareness of FMS [17] and find them difficult to apply in practice compared with general PA [18], children’s motor competence significantly improves when caregivers take an active role in programmes and are provided with clear instructions [19,20,21]. Such outcomes illustrate the importance of targeted messaging to educate caregivers about children’s FMS development and PA behaviours. In this context, family and behavioural factors within the home environment are recognised as important predictors of motor and behavioural outcomes in early childhood [22]. These influences are relevant to understanding risk trajectories within established developmental models of motor competence and PA [23], reinforcing the need for greater integration of family factors into early developmental research. Importantly, there has been little exploration of how FMS and the Chief Medical Officers’ (CMOs’) PA guidelines for young children are communicated to the public, and how breakdowns in communication and reduced movement opportunities represent modifiable determinants of later motor difficulties. This study helps to address this gap.

Despite increasing appreciation of the key role caregivers and families play in supporting children’s FMS during early childhood, the qualitative literature examining the determinants of family participation remains limited [17,24]. According to the United Kingdom (UK) Medical Research Council, combining qualitative insights from key stakeholders with quantitative evidence can strengthen intervention design [25]. Previous research in children’s PA has identified caregivers, educators, and children themselves as relevant stakeholders since they are most likely to be involved in intervention delivery and represent the target population [26]. This is equally applicable to family-based FMS research, which would benefit from combining insights from these stakeholder groups, alongside perspectives from subgroups whose voices are less frequently heard, to better understand families’ needs. Children’s perspectives warrant particular attention, as their preferences and lived experiences are frequently overlooked [27], meaning this study adds valuable learning relevant to future programme design which aim to support families.

Since children communicate differently from adults, creative and age-appropriate interview methods are necessary to enable them to articulate their thoughts [28]. One example of this is the Write, Draw, Show, and Tell technique, which is responsive to developmental differences in cognitive and communicative abilities [29], and has been used extensively to explore various aspects of PA in primary-aged children in the UK and Ireland [30,31,32,33,34]. But working with younger populations presents practical and methodological challenges [35], and so comparatively less research has focused on the early childhood period. Previous studies have successfully accommodated 4–5-year-olds by simplifying the method to draw-and-tell [36,37], although authors still deemed this to be unsuitable for 3-year-olds despite these adaptations, leaving their voices largely unheard. Young children also struggle to differentiate PA, movement, and play [36], which limits their ability to express views on FMS. Although a recent review used children’s experiences of active play at home as a proxy for FMS [24], their perspectives have not been reliably incorporated into the FMS literature. Therefore, there is a clear need to investigate early-years children’s perspectives on active play while cared for by their family, offering insights relevant to early FMS development and including children as young as 3 years of age.

The aim of this study is to qualitatively investigate the determinants of family participation in children’s FMS in early childhood, from the collective perspectives of educators, caregivers, and 3–5-year-old children. To understand children’s perspectives, this study will explore their active play preferences to identify their potential FMS development needs, while also assessing the feasibility of an adapted draw-and-tell methodology for this age group.

## 2. Materials and Methods

### 2.1. Study Design

In meeting the aims of this study, this research utilised an interpretivist qualitative study design to examine key stakeholders’ views on the determinants of family participation with FMS in early childhood. Interpretivism aims to understand the meaning that individuals attach to their actions and environments through a socially constructed reality [38]. This approach supports collaborative dialogue between researchers and participants, allowing the co-construction of a shared and meaningful understanding [39]. The interpretivist lens was therefore well suited to examining stakeholder perspectives on how families engage with early-years children’s FMS. Study reporting was in line with the Consolidated Criteria for Reporting Qualitative Research (COREQ) checklist [40] (see Appendix A, Table A1) to maintain transparency and comprehensive reporting of all study components.

### 2.2. Research Team

This research was conducted by a team of three white British researchers (female *n* = 1, male *n* = 2). The Principal Investigator (R.F.) was a PhD researcher with several years of academic experience, had prior publications in children’s FMS development, and completed additional training in qualitative research methods and interview techniques before data collection commenced. The project was overseen by the Director of Studies (C.R.) and First Supervisor (A.P.), both of whom had extensive experience in qualitative research relating to children’s FMS, PA and health.

### 2.3. Recruitment

Following ethical approval from the College of Science and Engineering Research Ethics Committee at the University of Derby (ETH2425-0144), a purposive convenience sampling approach was used to recruit participants. The research team aimed to recruit stakeholders whose views were not well represented in the previous literature, and to capture perspectives from as many groups that were closest to the issue as possible. Eligible participants were normally developing 3–5-year-old children, professionals involved in their care or education, and parents, grandparents, or legal guardians of children in this age group. Children with special educational needs and disabilities (SEND), carers of SEND children, and professionals specialising in the education of SEND children were excluded. Children with SEND are more likely to experience delays in motor development and in speech and language [41], and may find traditional interviews challenging unless carefully adapted to meet their needs [42]. To avoid these factors negatively influencing adult perspectives on FMS, hindering children’s interviews, or causing distress, these groups were not considered for the purposes of this study.

Participants were recruited through existing professional networks, early and primary education settings, and local community groups via email, social media, and word of mouth. Adult participants were recruited from Derbyshire in the East Midlands of England. Further participants were recruited nationally to add greater geographical and socio-economic diversity to the sample. A range of professionals with different job roles, responsibilities, and experiences of working with 3–5-year-old children and their families were intentionally targeted to add greater depth and ensure a variety of perspectives were captured. Children were recruited locally for practical, ethical, and logistical reasons for both the researchers and participating families.

### 2.4. Participants

A total of 34 participants took part in the study. The final sample included 15 primary caregivers that consisted of 11 parents (female *n* = 8, male *n* = 3) and four grandparents (female *n* = 2, male *n* = 2). A further three parents provided informed consent but did not take part in an interview and were subsequently withdrawn. Eight educators participated, including two female generalist primary school teachers, one female Early Years Foundation Stage (EYFS) teacher, one female EYFS teaching assistant, one female nursery playworker, one male PE specialist, and two family hub practitioners (female *n* = 1, male *n* = 1). Family hub practitioners are outreach workers based in government-funded children’s centres and offer parenting advice, education, and support to families with children from early childhood through to adolescence, including children’s physical development [43]. Family hub services are promoted locally through healthcare, education, and community settings, which also play an important role in referring families who need additional support to these services. The research team became aware of this group early in the recruitment process and recognised that their unique role in family education and early physical development had been overlooked in the literature. Therefore, family hub practitioners were specifically targeted to ensure their valuable insights were represented. Finally, twelve children initially took part in the interview activity. However, one child was withdrawn because they were unable to satisfactorily complete the task, leaving a final sample of 11 children (female *n* = 7, male *n* = 4).

According to the English Indices of Multiple Deprivation [44], participants represented a heterogeneous spread of socioeconomic backgrounds, as shown in Appendix B, Table A2. These indices relatively rank each small area of England from most to least deprived (1 being most deprived and 32,844 being least deprived) using seven domains of deprivation including employment, income, health, education, barriers to housing and services, crime, and living environment.

### 2.5. Instrumentation

For adult participants, data were collected through individual semi-structured interviews, which are well suited to encouraging open and honest accounts and generating rich personal depth in participants’ experiences [45]. The interview schedules were developed using findings from previous work conducted by the research team [24] and were further guided by the relevant literature [17,18,46,47,48]. For children, interviews were carried out using an adapted draw-and-tell methodology [36,37]. Due to the historical challenges of applying this method in 3-year-olds, a major objective of the current research was to further refine the technique so that meaningful contributions may be obtained from this population as well as from 4–5-year-old children. In recognition that very young children are still developing their representational drawing skills, previous children’s participatory research studies have recommended adopting flexible, child-centred approaches when applying drawing techniques in this group [35,49]. For example, greater emphasis should be placed on children’s verbal explanations rather than trying to interpret ambiguous or unidentifiable drawings alone [49], and incorporating playful activities can encourage conversation with those whose responses may otherwise be narrow or limited [35]. These concepts therefore guided development of the methodology to work towards the full inclusion of 3-year-olds.

Three separate interview schedules were designed to allow for the differing roles of caregivers and educators, and to account for children. These schedules are available in Appendix C. Despite caregivers and educators having separate schedules, the overarching aims and objectives remained the same, with questions that intended to (1) establish awareness and understanding of FMS and the UK CMOs’ PA guidelines for children; (2) identify key perspectives on the determinants of family participation in FMS during early childhood; and (3) understand interventional needs and preferences for families. While children’s questions were constructed to (1) ascertain activity preferences; (2) understand what makes PA enjoyable to them in the home and community; and (3) determine what factors prevent or support children’s PA participation when in the company of family. Piloting of the questions took place on a small number of individuals similar to the target population to determine what worked well and what refinements were required to achieve desired information and to ensure that participants felt comfortable. Questions were open-ended and responses were probed to facilitate comprehensive offerings from the participants. The interviewer also asked additional questions outside of the interview schedule to encourage elaboration on noteworthy points that were raised. Discussions between the research team were held at regular intervals to confirm consistency in the interviewing approach and to maintain robustness of the research processes.

### 2.6. Procedures

All interviews were conducted by R.F. between December 2024 and April 2025. Upon receiving informed consent, participants were invited to take part in one individual semi-structured interview. As interviews commenced, R.F. introduced themselves and explained the purpose of the study. No personal motivations or assumptions were shared, and a professional, neutral stance was maintained throughout. To accommodate differing backgrounds, needs, and preferences, adaptable interview formats were offered to improve engagement [50]. Participants could choose to interview face-to-face in a mutually agreed, safe, and appropriate location in the community, or via Microsoft Teams. This allowed data to be collected nationally and helped to remove potential barriers relating to family or work commitments, time constraints, cost, and accessibility. The average interview duration was approximately 58 min. No repeat interviews were conducted.

For children’s interviews, following receipt of written caregiver consent, children were asked to provide assent to take part in a draw-and-tell activity. All interviews were completed face-to-face for practical reasons. Previous work [37] recommended conducting interviews in child dyads to minimise potential intimidation for participants. However, children could not feasibly be interviewed in pairs when families were met individually. Moreover, the research team held concerns that this approach may encourage copying, and so children were interviewed one-on-one in the presence of a trusted adult in the community or in familiar community groups to ensure children felt safe and comfortable. R.F. also spent time interacting and playing with the children before commencing the task to build trust and rapport, as children may become shy and withdrawn around unfamiliar people [36].

Once assent had been obtained, children were asked to draw a picture of themselves engaging in their favourite play activity at home. While children were still actively drawing, they were invited to explain what they were drawing and why they enjoyed the activity, enabling spontaneous and authentic conversation without making children feel tested or pressured [37]. If children had chosen a sedentary activity, this was noted as their preference, but they were then redirected to examples of PA. Following this, the children were asked a range of questions that explored what supported or prevented their participation in active play in the home environment, and what made their play enjoyable. It was anticipated that 3-year-olds were unlikely to draw anything identifiable. Therefore, less emphasis was placed on the drawings themselves, with greater focus on what the children articulated them to be, as young children often give meaning to their marks or scribbles, describing them as people or objects [49]. Child-led play, using immediately available resources such as a community centre play corner, was also incorporated into 3-year-old interviews to maintain engagement and support their expression of ideas if children strayed from the original draw-and-tell task. This allowed the interviewer additional time to continue conversations with the children while they played. The average interview duration was approximately 20 min.

Face-to-face interviews were audio recorded using a digital voice recorder and were transcribed verbatim by R.F. Online interviews were recorded via the record and transcribe function on Microsoft Teams. If any participants had declined to be recorded, field notes would have been taken. However, permission was provided by all adult participants and caregivers. Transcripts were not returned to participants for comment or correction, as follow-up was not practical for children and online participants who were not local, and the research team was satisfied with the data and its meaning. In line with Fusch and Ness (2015) [51], data were collected to the point of saturation, where the data became repetitive and no additional information or insights were emerging that offered anything significantly different to the understanding of the topic.

### 2.7. Data Analysis

The interview transcripts were analysed using Braun & Clarke’s (2006) [52] six-stage iterative framework for thematic analysis, with themes derived inductively from the data and managed manually without the use of additional software. Thematic analysis is a widely recognised, valued, and flexible approach for identifying, analysing, and reporting emergent patterns and themes within qualitative data [53], and has been extensively called upon in qualitative research related to children’s FMS development [47,54,55]. Firstly, familiarisation with the data took place by repeated reading of the transcripts. Key sections were highlighted, and initial descriptive codes were assigned to capture interesting features in line with the aims and objectives of the study. These codes were collated into potential themes based on recurring patterns and similarities across the data (see Appendix D, Table A3), which were reviewed on multiple occasions to confirm they accurately reflected the underlying meaning. Clear definitions and names were developed to produce a final set of themes and subthemes that were illustrated in the results using relevant examples and quotations. Analysis was conducted by R.F., with regular meetings held throughout the process to reach consensus with the remaining members of the research team on the themes and their accuracy and trustworthiness.

## 3. Results

### 3.1. Adult Participant Characteristics

A total of 23 adults (female *n* = 16, male *n* = 7) participated (Table 1). Participant age ranged from 18 to 34 years (*n* = 5), 35 to 54 years (*n* = 12), and 55 years and over (*n* = 6). The mean age of the participants was 43.7 years (SD = 15.4). Most participants identified as White British (*n* = 22), and one participant identified as Black British Caribbean. Of the 23 participants, 11 were parents (female *n* = 8, male *n* = 3), 4 were grandparents (female *n* = 2, male *n* = 2), and 8 were educators with varying job roles. Participants were recruited from multiple regions, with 12 from Derby, 7 from surrounding Derbyshire areas (South Derbyshire, Northeast Derbyshire, and Amber Valley), and smaller numbers from Wigan (*n* = 2), Barnsley (*n* = 1), and Coventry (*n* = 1) (Table A2).

### 3.2. Child Participant Characteristics and Activity Preferences

A total of 11 children aged between 3 and 5 years (female *n* = 7, male *n* = 4) completed the draw-and-tell task (Table 2). The mean age of the sample was 4.5 years (SD = 0.8). Most of the children lived in Derby (*n* = 5) and regions of Derbyshire (South Derbyshire, *n* = 3; Northeast Derbyshire, *n* = 2), while one child was based in Leicester (Table A2). There was an almost even split between children who preferred active play and those who preferred sedentary activities (*n* = 6, 54.5%). It is important to note that some sedentary activities may alternatively be interpreted as active depending on how the child engaged in this play, but were subjectively determined based on the child’s description

**Figure 1:**
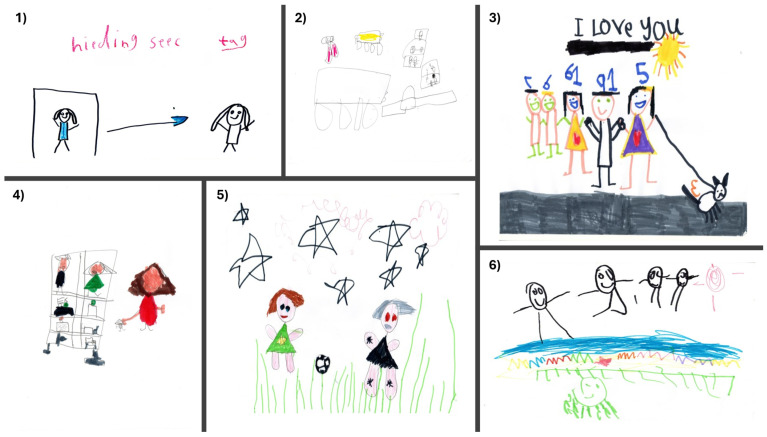
Examples of 4–5-year-olds’ drawings of their preferred activities. (**1**) Hide and seek tag with a friend (CH11). (**2**) Playing with toy cars (CH4). (**3**) Playing dress-up with family (CH8). (**4**) Playing with dolls (CH6). (**5**) Playing football with mum (CH7). (**6**) Swimming with friends (CH1).

**Figure 2:**
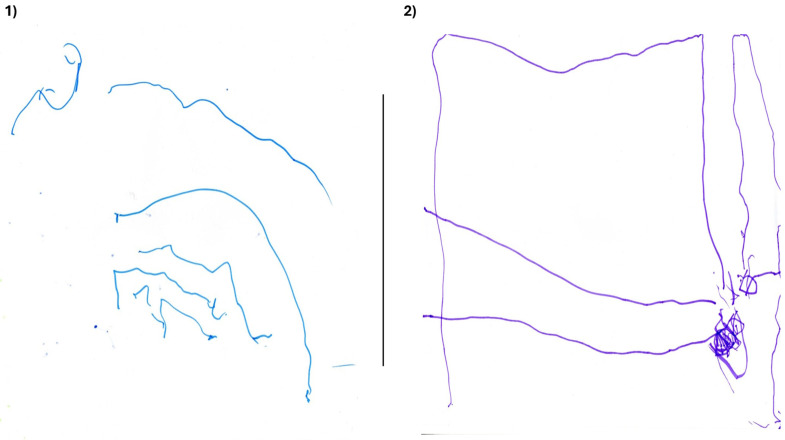
Illustrations by 3-year-olds. No identifiable features were drawn. Interpretation was gained through their articulation of the drawings and by play-based sharing. (**1**) Swimming with family (CH3). (**2**) Playing with dinosaur figures (CH9).

The drawings produced by children varied considerably in clarity and detail. Some children clearly represented their favoured activities, whereas others produced drawings that required further discussion to interpret. For example, item number 6 (Figure 1) was initially thought to be a picture of the child with their family at the seaside. However, it was subsequently revealed that the drawing represented swimming with friends. As expected, 3-year-olds’ drawings contained only unstructured markings (Figure 2). However, these children were able to verbally explain what their drawings were, and the playful adaptations used during the task helped maintain children’s engagement for longer periods, enabling extended conversations about their thoughts and feelings regarding play.

### 3.3. The Determinants of Family Participation in FMS

**Figure 3:**
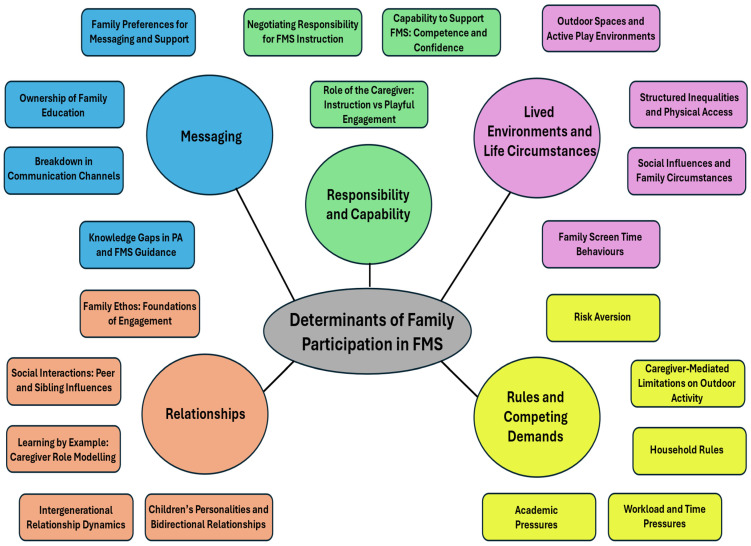
Thematic map from thematic analysis. The research question appears centrally within a grey oval, with the five main themes branching outward in coloured circles. Subthemes are presented in matching coloured rectangles adjacent to the corresponding themes. FMS: fundamental movement skills; PA: physical activity.

The data compiled from educators, caregivers, and children were synthesised during analysis, resulting in five main themes relating to the determinants of family participation in FMS: messaging; responsibility and capability; rules and competing demands; lived environments and life circumstances; and relationships. The themes and associated subthemes are presented below (Figure 3). In the text, participant quotes are labelled to indicate stakeholder group followed by participant number (PT = parent; GP = grandparent; TE = teacher; TA = teaching assistant; NW = nursery worker; PE = PE specialist; FP = family hub practitioner; CH = child. During interviews, participants referred to FMS, PA, and play interchangeably when discussing issues related to FMS. Given the qualitative nature of the research and the importance of representing participants’ perspectives accurately, the following results section reflects the terminology used by participants in their accounts.

### 3.4. Messaging

#### 3.4.1. Knowledge Gaps in PA and FMS Guidance

Awareness of the UK CMOs’ guidelines for children under 5 years and 5 to 18 years was poor. Only two caregivers were able to accurately recall any of the core principles of the guidance:

“[Children under 5 years] *180 min per day, and it is all about moving more, being active, being stronger….* [children 5 to 18 years] *60 min per day, and it is the same principles, like less sedentary time, making sure they are moving more through a range of activities.*”

(PT4)

“*Yes, 180 min per day with 60 min of moderate to vigorous physical activity for the under 5 s and 60 min per day for 5 to 18-year-olds.*”

(PT3)

None of the remaining participants could correctly describe the PA guidance, and this lack of knowledge extended to all of the educators in the study:

“*I don’t really know. I know there might be information that people or groups put out for the little ones. But what the actual guidelines are, I wouldn’t know if I’m honest.*”

(GP3)

“*I’ve not looked at it for a while. At a guess I think it is something like two hours per day, or something like that. But I haven’t looked at it for years.*”

(FP2)

“*No…but can I just say that I took a step back from being a nursery nurse a few years back and am just acting as a playworker now, so I don’t go to any of the professional meetings. I’m not involved with it, and I’m not expected to know these sorts of things.*”

(NW1)

Participants were subsequently asked about FMS. Overall, awareness and understanding was limited, though a few could discuss the concept confidently:

“*That is your balance and being able to climb…being able to catch a ball and activities requiring coordination, like maybe doing an obstacle course where you would need to weave in and out and keep your balance. It would be all your core skills and using your larger muscles.*”

(PT9)

“*They link with sports and activity when you get older… learning how to throw, catch, kick in that 3–5 years age bracket… will then prepare you for those sports-specific skills. You build those fundamentals in the first place so that they can move onto more complex stuff later.*”

(PS1)

However, most participants offered narrow, unconvincing, or inaccurate descriptions of FMS, and many had never heard of the term before. Once again, this included the educators:

“*I think they are what they are doing physically with their hands, knowledge of things, talking. When they go outside, they are things that they notice.*”

(GP2)

“*No, I haven’t heard of that before.*”

(TA1)

Some participants suggested that FMS would be better understood by early childhood specialists, and that the term “gross motor skills” was more widely recognised:

“*I think early-years teachers would be more aware of it, but if you were a primary teacher and you didn’t specialise in PE, then I wouldn’t be sure how much they would know. I don’t think parents would know what they are. Not unless they worked in that sector.*”

(TE1)

“*I think there needs to be a little bit more support or education for parents… because if I asked parents to give me six fundamental movement skills, I don’t think they would be able to say. I don’t know, maybe if it were gross motor they might be able to name a couple.*”

(TE3)

Indeed, participants who were unfamiliar with FMS were alternatively asked, as planned, to describe “gross motor skills” instead, which drew noticeably stronger responses:

“*Yes. So, they are your jumping and skipping and all those sorts of things that they are meant to do. Maybe balance and co-ordination too?*”

(PT5)

“*Gross motor is the larger movements like running. I always see it as being more with your arms and legs, because at school we say that gross motor is all about the bigger movements.*”

(TE2)

Participants felt that terminology was inconsistently applied and differed between research and practice, contributing to less recognition of FMS compared to “gross motor skills”:

“*I think fundamental skills are more of a research term. Whereas as early practitioners we were always trained to use the terms gross and fine motor, and I think it is an easier thing for parents and families to understand as well.*”

(FP2)

“*I think when you coin it FMS you segment it from gross motor skills… FMS allows you to categorise these gross motor skills… I know that there was some discussion around them being called core skills instead of FMS… with terminology we need to be clear from the outset, and it might be that for educators we just use the term gross motor when speaking of FMS.*”

(PT4)

#### 3.4.2. Breakdown in Communication Channels

Caregivers consistently felt that guidance on PA and FMS was failing to reach families. Several described having no previous exposure to any guidelines before taking part in interviews:

“*This is not something as a parent I have heard of. I know you have just told me that guidance is available online, but no, this hasn’t got to me.*”

(PT5)

“*I would say it isn’t communicated well. I wouldn’t have even known about it all until I did this interview with you today. So no, I don’t think it is something that people know about.*”

(GP4)

Participants explained that families were not receiving guidance through the intended communication channels across education, health, or the community:

“*The information is out there, but it isn’t readily communicated by anyone. You don’t really get any communication on this subject from nurseries or schools that they should be doing this skill or that amount of activity… I don’t think that is put across. It’s not advertised anywhere.*”

(PT10)

“*Back in the day there was a lot more information and support for parents when there were the SureStart centres* [some of which now operate as family hubs] [43] *… but these places are disappearing now, and a lot of parents are not getting to learn about the importance of physical development.*”

(TA1)

“*We do have nurses and healthcare workers come into school… but they don’t seem to do anything to promote this. They go to houses to safeguard and come in to do nasal sprays and that, but we’ve not really had them for anything else. So, there is potentially a gap there.*”

(TE2)

Participants highlighted limited FMS support from health visitors. Support in general would also abruptly end after 2 years, leaving them feeling unsupported at a crucial stage of development:

“*No, I can’t recall them mentioning fundamental skills. I definitely know from my own experience with health visitors that it isn’t something that they necessarily sit and talk to you about. They do that 2-year review with your child, and you fill out that form and you have your red book… but I would say in terms of your fundamental skills, I don’t recall that at all.*”

(TE3)

“*Our health visitor saw us a few times, but they last saw my son when he was still 2. It was a tick box to say he was toilet trained and that, and then we have never seen or heard from anyone again… There is no more guidance coming through. Not even a letter through the post. There is no information on what we should be looking for and how and why we need to be promoting it.*”

(TA1)

Participants criticised health visitor input and wider messaging as reactive and diagnosis-orientated, instead of offering positive guidance or proactively promoting PA or FMS:

“*The only time anything was communicated to us was when she wouldn’t walk when she was younger and the health visitor wanted to send her for x-rays. But then she just stood up and walked anyway. Once she started walking that was the end of that and no more communication. It was well over a year ago. If she wasn’t walking right now, they would be all over it.*”

(PT6)

“*They go on about obesity in children… They don’t focus on the things that could actually help them. For instance, physical things that you can do with your children… I know how the schools measure BMI and tell you your kids are fat or overweight… Children might become anorexic or have problems with their eating. Don’t tell them they are fat.*”

(GP2)

“*There should be more positive messages from the powers that be. There is too much onus on the problem and not enough on the solution… It would be more helpful if we had more information on what parents could do as opposed to just going on about the problem. We need more positive encouragement and ideas*”

(PT3)

Participants also reported on how healthy eating was more actively promoted than PA and FMS guidance, creating uneven family awareness and understanding of the topics:

“*We tend to get more on the healthy eating side of things and I’m more aware of that. Everybody knows you are supposed to eat 5 a day and stuff like that. But I don’t think the physical side of things is as well known and in general I don’t think it is very well communicated.*”

(PT9)

“*They used to send leaflets home about your diet, and there used to be a lot of communication about you eating correctly. But in terms of the physical side of it, perhaps not.*”

(TE3)

Participants stated that families were unlikely to seek guidance if they were not first made aware of it, leaving key information effectively invisible. Therefore, they emphasised the need for clearer signposting to raise family awareness and facilitate access:

“*I wouldn’t think parents would go and search on the government website. I don’t think it is a normal thing. Especially if they haven’t heard of it. Why would they search for it?*”

(PS1)

“*If you don’t know about it and no one is pointing it out to you then how are you supposed to know? There are millions of pages on the internet. What are the chances of you stumbling on the right page if you don’t even know you are searching for it? It has to be conveyed to the parent.*”

(PT6)

“*Families are not going to just go and search for information on the website… It’s all down to signposting and getting down to that level to them.*”

(FP1)

“*I think it is a resource that you should be signposted to so that you know it is there, but not one that you are forced to read. You don’t necessarily need everything printed out in front of you, but you should have it available to you and be reminded of it.*”

(PT9)

#### 3.4.3. Ownership of Family Education

Participants expressed differing views on whose responsibility it was to improve family understanding of FMS. Some believed that it was parents who held primary responsibility:

“*I think first and foremost it’s me. It’s my responsibility because I am the parent and they are my children.*”

(PT5)

“*I mean, speaking from my own point of view. I’ve got two children of my own. No one ever told me what you’ve got to do or how you do it or where you take them. It was just instinct and using my own initiative to find out.*”

(NW1)

Grandparents expanded on these views by describing themselves as a source of intergenerational knowledge who may contribute to family education on FMS:

“*Can I just say that I was a parent once and brought up my own children. I think grandparents are such a big part of children growing up now. You can learn from grandparents too. So, I think it should be the parents and the grandparents.*”

(GP2)

“*I think this is actually something that can be passed down. I learned a lot from my parents and hopefully our children and grandchildren have learned a lot from us. It is the sort of thing that should be shared through the generations. That motivation to be active and mobile, to be outside with fresh air in your lungs. It is so important and should be passed down.*”

(GP1)

“*I think it comes down to experience because we have been there, done that, and got the t-shirt already, and it should be passed down. I think it needs to be passed on to the parents and then the grandchildren.*”

(GP4)

Other participants argued that schools and nurseries should provide caregivers with educational support on FMS through existing communication channels and alongside academic reporting:

“*I would like to know more from school about what we should be working on at home. It should be communicated along with academic reporting. Yeah, schools need to do more.*”

(PT1)

“*I think schools would absolutely be the right way to do that. You could ask schools to stream this information. Put it on our website, put it on messaging platforms, on Dojo. Things like that.*”

(TE1)

“*It should come from nursery. We will talk about where they are in terms of various bits of development. But they have never mentioned gross motor skills or what the recommendations and requirements are. So, I think they could quite easily add that in and that would help parents.*”

(PT8)

Conversation on the ownership of family education prompted one educator to reflect on what their own school could do to improve support for families and raise awareness of FMS:

“*Perhaps our PE lead could sit down and deliver a workshop and go through this with parents so they are more aware of how they can help at home. Because we do it for maths and English, but we don’t necessarily do it for the physical development.*”

(TE3)

Echoing this enthusiasm for collaboration, several participants believed that strong caregiver-educator relationships were the key to improving family understanding of FMS:

“*I’d say more than anything, it is the responsibility of the parents to make sure they know these things. But I guess it would also need to come through teachers at school, because I don’t know how else I could get exposed to this. So, I certainly think it needs to be between the two.*”

(PT2)

“*I think we have the closest relationship to parents, and I think it is like teamwork between schools and parents, rather than relying on someone you don’t really know, like in the health system. That might feel a little threatening to them. Whereas I think we have a closer relationship because we are working directly with the children.*”

(TA1)

“*When I have worked in schools I have seen a lot in the way of teacher-parent relationships. They even sometimes call each other by first name. So, I feel there is an opportunity there to get this across to parents through this relationship. I think parents need that base of knowledge to even know that they need to research it. They get that base from professionals, then the responsibility passes to the parents.*”

(PS1)

But participants largely agreed that responsibility for promoting FMS in early childhood is shared between policy makers, educational settings, the health sector, and caregivers themselves:

“*I think it’s everyone’s responsibility to improve family understanding of FMS. From the top down, there needs to be more. The early years is where outcomes should be most intertwined, and all parts of the jigsaw should all fit together.*”

(PT3)

“*I think it needs to be multi-pronged. It is about providing that information to parents and offering guidance to help through nurseries, family hubs, children centres, outreach workers. So that parents are getting the right information from several avenues and are feeling supported.*”

(FP2)

“*With this particular age range, I think some communication has got to come from the health side through the health visitors and so on. But also, from your educational settings. From nursery upwards. From the parents themselves… it has to be an amalgamation of everybody.*”

(TE3)

#### 3.4.4. Family Preferences for Messaging and Support

Participants were invited to share their views on families’ educational needs and preferences for FMS support. Some felt that face-to-face communication was the most effective approach:

“*I think you can get a lot more across face-to-face compared to sending them a text message or sticking it on the internet. I think face-to-face contact is really important*”

(PS1)

“*I take in much more information from physically seeing and hearing it in a workshop or class. For me personally, I get distracted watching a video or something at home, but that’s just me.*”

(PT5)

Grandparents favoured face-to-face workshops held at times and in locations, such as schools and community centres, that would support caregiver attendance:

“*I think it could be done in connection with school. Some form of activity evenings. It doesn’t necessarily have to be the parents that go. It could be the grandparents… If you made it immediately after school, then parents or grandparents are always going to be there to pick the children up, so it would be a good time to do it.*”

(GP1)

“*I would say get it out there in the community in a big room. Somewhere where the family could go and let the kids have fun doing it. I think it needs to be a two-way thing. By all means put it on the internet but it’s better being human beings and getting it out there.*”

(GP4)

However, some participants questioned the feasibility of these workshops, arguing that digital communication would be a more realistic alternative due to its comparative accessibility:

“*I prefer a workshop. But based on my own experience, workshops in the past have hardly been attended. Maybe because parents are working. People are at work more now.*”

(TE3)

“*I always say the best way to learn is in a classroom setting. Someone physically showing you… But if that wasn’t possible then videos would work… With families constantly trying to cram everything in, you might not always have time to go to classes, so utilising the internet makes sense for working families.*”

(PT2)

Indeed, many participants strongly preferred digital FMS support, highlighting the convenience of video demonstrations, and the enjoyment and greater privacy that this approach provided:

“*If you wanted to reach families at home, then an app. The app would get you into the home…It is the way forward and what the families respond to. They all have tablets and mobile phones.*”

(FP1)

“*I like the idea of using an app, but it takes you ages to log in, find an activity, and watch it, and by that time my 3-year-old has run off because she’s fed up. So, I would rather watch something in my own time and then play the game with them the next day as opposed to resources that encourage you to do something with your child there and then. You could put it on YouTube.*”

(PT3)

“*We use Danny Go Exercise Physios on YouTube. We have that on in the evening sometimes and the children use it to move. They will do a couple of different workout videos, and it is all sort of fun songs and dance that are aimed towards children. You have got them active using tech.*”

(TA1)

“*Something like YouTube would be brilliant. You could be given videos to watch, and it would probably be easier for parents, especially those who struggle to get involved physically. Also, it perhaps wouldn’t be quite as daunting as going into a class where you might feel under pressure to take part. I think socially parents feel that apprehension as well… So, I think something you can do at home in private as a family.*”

(PT10)

Caregivers called for brief, simple, and easy-to-digest resources to help maintain children’s attention and avoid overwhelming families:

“*When your children are young you get so much information thrown at you. Too much information. It can be really overwhelming sometimes because you end up with various packs with all these different leaflets.*”

(PT5)

“*As parents you don’t want reams of paperwork to have to go through. So, whatever the information is needs to be bite-sized so that it is easier to digest and you can take on board whatever is on there.*”

(PT9)

“*Children would get bored after a bit. Like if we do Cosmic Yoga on YouTube, they will watch 10 or 15 min and then they are done. But if it was bite-sized support, then yeah. I think if the message could be got across quickly, however that may be, at home, then that would be far easier because you could just balance it around whatever you have got going on in your family life.*”

(PT8)

Some participants also suggested targeting children with messages, and including them in activity design to enhance their engagement:

“*Maybe you could have subtle little messages for children alongside the parents. Because if the children can grow their understanding too on how that’s important for their bodies, and how they’ve got to get up and move, then a lot of children will take that on board, won’t they.*”

(FP1)

“*You could even get the children to help design the activities and be involved in it, putting their own ideas in. Because then they have had their say on what they want and are actually telling you what they want. I think that would be good because they all have different needs.*”

(GP2)

### 3.5. Responsibility and Capability

#### 3.5.1. Negotiating Responsibility for FMS Instruction

Participants agreed that FMS development was a shared responsibility between educators and caregivers, but views differed in how responsibility for supporting skills should be divided:

“*Teaching of the skill should probably stay within an educational setting…professionals should be teaching those technical aspects. The basics of the movement should be supported by parents, but then to get the technique right, that should be through the educators.*”

(PS1)

“*I think ultimately it is our responsibility as parents to help our children develop. But obviously when they are in a nursery setting you would expect them to support it as well. So, primarily with us, but then for the time that they are there, then nurseries should support as well.*”

(PT8)

“*I think it is an element of both, because it needs to happen in settings and then be practiced at home. If you only do it within a setting, they’ve only got that element of being able to practice whilst in that setting. If you can educate parents and get them on board, you have potentially got a bit of both… because you could say it’s a massive period of time with the families, isn’t it.*”

(FP1)

Nevertheless, underlying tensions emerged as some caregivers felt that responsibility should shift primarily to educational settings once children attended nursery or school, while others felt schools placed too much expectation on families:

“*Basically, it is part of physical education. So, as soon as they are at nursery, then they should be teaching it there. Initially, it is on the parents while they aren’t at school. But when they do start going that is when the physical education side of things should begin.*”

(PT6)

“*In school* [for FMS teaching]. *I do think parents and family have a duty to do it, but it’s almost as if schools expect parents to take control of the physical side of things. Children should mostly be doing this at school so that at home you are only going to be giving them further advantage. School’s definitely need to take on more.*”

(PT1)

Whereas educators believed families should play a more active role in FMS development and opposed some caregiver views that support should fall predominantly on educators:

“*I 100% think parents need to take more responsibility. Schools and education can’t do everything. We’ve seen them for six hours in the day, but there are 24 h in a day. We need help filling the gaps in the PE curriculum. So, I think parents also need to step, generically.*”

(TE1)

“*They think it is all our job as educators to teach this. They think we are the ones who are supposed to be doing this role, but actually it’s not because you they are the parents at the end of the day. We facilitate so much, but we can’t do everything.*”

(TE2)

Some caregivers were comfortable with educators taking on additional responsibility for FMS support, trusting that teachers were trained, qualified, and capable of delivering this:

“*I’d like to think they are qualified and confident. It’s not all about reading and writing. It’s just as important for teachers to teach the physical side and provide outside time and encouragement.*”

(GP2)

“*My assumption is that it is in their training and their updates and standards.*”

(PT8)

These assumptions were challenged by certain participants who revealed that educators are not always well positioned to effectively teach FMS:

“*I feel PE is not publicised enough within education while you are training. So, I feel ECTs* [Early Career Teachers] *lack knowledge and confidence. When I’ve had student teachers, that’s the lesson that they are most scared about teaching, and they feel they lack the experience.*”

(TE2)

“*The newer teachers only had one afternoon of PE training unless they were specialising in PE… A lot of teachers are coming into teaching learning on the job and learning from other teachers who are not confident in PE and are not equipped to deliver and teach those skills.*”

(PT4)

“*I feel as though other things just get introduced all the time and then something else has to slip to accommodate. There are not enough hours in the day to teach all these things.*”

(TE3)

“*Some people in education do not like PE and therefore they’ll get out of doing it, when actually that is the one lesson that the children definitely need. PE is always the first thing to go.*”

(TE2)

#### 3.5.2. Capability to Support FMS: Competence and Confidence

Participants considered how competence, capability, and confidence shaped caregivers’ support of FMS. Several noted that high personal skill was not required for basic FMS support:

“*I don’t think you have to be brilliant at any of those things. For children of that age, it is more important that you are just there with them doing it and encouraging it. If they can see you making mistakes, that is a lesson in life that we are not always going to be the best at everything.*”

(TE3)

“*It would be a help* [caregiver competence], *but that’s not a barrier. I don’t care if I am inept at catching. I can still encourage her to catch. We are talking about 3- and 4-year-olds here… You are just catching and kicking or rolling a ball*”

(PT3)

Whereas some participants argued that caregiver competence was necessary, believing that without it, caregivers could not demonstrate or guide children through practice appropriately:

“*Yeah [caregivers need to have competence]. So that they can actually show them what to do and to help them do it. You’ve got to show them how to do it, haven’t you.*”

(NW1)

“*I feel they probably do* [caregivers need competence]. *Because in my coaching, if I couldn’t kick a football then I wouldn’t be able to teach someone else how to kick a football… If you can’t do something, then are the children going to take any notice? They aren’t setting the example.*”

(PS1)

Participants recognised that for some caregivers, health or mobility could restrict their involvement in children’s activities, limiting their capacity to model skills directly:

“*Parents might have a disability that stops them taking part. If you have a parent who can’t walk, that would make it harder to be physically involved or to teach their kids how to kick a ball. You could teach the theory, but it would make it difficult to physically teach in these situations.*”

(PT9)

“*I’ve got heart issues. So, it does restrict me in what I do and what pace I can do it. If he is running about, I can’t keep up with him. I’m not going to get any more mobile than I am now. You know your own limitations when you get older and you know you can’t do certain things.*”

(GP4)

However, several participants felt that physical restrictions should not prevent caregivers from providing active experiences for children through indirect support and creative strategies:

“*I have fibromyalgia and arthritis. Physical activity isn’t something I’m always capable of. I have no grip in my hands. So, if the children wanted to play catch with me then it wouldn’t be a very good game. But that doesn’t mean I will stop them doing anything. It would be ‘right, how can we tackle this?’. I might get them to show me what they’ve done at dance or put some music on. It’s not a lot of involvement from me but they are still doing things themselves.*”

(PT10)

“*I worked with a family with a dad who had physical disabilities, but it doesn’t have to be a limitation. He went around on his mobility scooter and got that child playing football and that. He couldn’t run with him, but he was still encouraging physical development through fun. He was cheering him on. He was present, empowering his child, and having fun with him.*”

(FP1)

Although physical capability was acknowledged, participants highlighted that confidence and attitude may be more decisive in whether caregivers engage with or avoid certain activities:

“*If you struggled to throw and catch, then you probably wouldn’t want to do it yourself. You just wouldn’t choose that activity to play. You would do something you are more capable of. But that would mean your children don’t get to do it. Or maybe you could use that as an opportunity to do it better yourself and learn at the same time as them. It would depend on your attitude.*”

(PT8)

One parent continued by reflecting on their own sporting ability, which had provided the knowledge and competence to confidently guide and transfer skills to their children:

“*I would say it* [confidence to support] *stems from being active when I was younger. I played football for a number of years. I mean, I don’t know what I’m doing in every skill. But a lot of my confidence is by having that experience myself and having observed other people doing these things. In general, I know the right and wrong way of doing stuff.*”

(PT2)

Comparatively, participants described how a lack of confidence could compromise their engagement with children, even with a strong background in sport and PA:

“*I would never go out for a run with my children because I’m not very good at it and I don’t want them to see me fail. You want to be seen as a superhero in your children’s eyes.*”

(PT9)

“*I would say I have the confidence to facilitate free play or physical activity, but perhaps not to teach specific skills. Even though I have worked in sports and physical activity, the moment I was faced with a toddler I had absolutely no idea what she was supposed to be doing. So, for someone who has not got my experience, how are we expecting them to do it?*”

(PT3)

One family hub practitioner argued that parents were more capable than they realised, and with gentle reassurance could feel more comfortable supporting children’s FMS:

“*For some of them, it is parents’ own perceptions and thinking that they can’t do this. Sometimes what we find in our roles as family visitors is that it is not us telling them how to do things; it is us supporting the family to realise that they potentially already know how to do this and making them feel comfortable. As soon as you mention something, they will go, ‘oh yes, we could do this, and we could do this as well.’ You need to help them to flick the switch.*”

(FP2)

#### 3.5.3. Role of the Caregiver: Instruction vs. Playful Engagement

One parent was unsure if caregivers should be teaching FMS or simply supporting unstructured free play. They linked this uncertainty to a conflict in messaging on the topic:

“*There is a conflict in messaging. What do you want us to do as parents? Do you want us to help teach or just get them out there and play? There are two schools of thought on whether this should be play-based or if we need to teach them FMS in a structured way.*”

(PT3)

Many participants felt that caregivers should be prioritising free play and supporting enjoyable, everyday activities rather than deliberately teaching FMS to children:

“*In this age group we are talking about play-based activities… The idea of play is taking part in an activity with no end result. So, literally going out and jumping in a muddy puddle… Some of my favourite memories from when I worked in the nursery were water fights… It was infused in the play, and this should certainly be the case at home too.*”

(FP2)

“*Teaching I find is quite regimental. You are telling them to do this and that in a certain way. Whereas play is more natural and I would hope they are happier. You reflect on some of the fun stuff you did yourself as a kid like building assault courses, and it is this natural type of fun you can have with your own children.*”

(PT10)

“*I don’t think we should be asking families to follow a programme. We should be teaching motor skills through things like discos and dance, or by doing tuff trays, picking up rice and things. They don’t know they are learning because it is discreet. In my opinion, the role of the parent is less formal. They should be doing these things through very informal approaches.*”

(TE1)

Some caregivers described teaching occasionally, but only when this was absolutely necessary, intervening only when a child struggled. Play would be otherwise left uninterrupted:

“*If we were doing something where I could say ‘oh, if you do it this way it could be better’, then yes, I would intervene in play and teach them. But only if I thought it was necessary because if they were enjoying what they were doing then I probably wouldn’t disrupt that.*”

(PT1)

“*I don’t think you do it consciously* [teach FMS]. *You just have fun and play and interact with the children. We don’t teach. We just play. If he was struggling or doing something wrong while trying to catch, we would try to guide him… Show him the right way and praise him when he catches it. But I think you do it naturally when you are playing mostly, and you don’t even realise.*”

(GP4)

Although play-based learning was widely valued, some participants recognised that deliberate guidance was occasionally needed for more technical skills, even when incorporated into play:

“*I would say we just do general free play. But she likes to practice her gymnastics so we might work on her form and balance if she wants to work on her handstands. That’s when we would take a bit of time out of play and try and work on and encourage those skills.*”

(PT5)

“*We do practice things like when they are playing football and getting them to shoot into a goal. So, aiming. Throwing and catching we have practiced. So, there is some structure there, but you are still kind of incorporating it into a game.*”

(PT8)

However, one educator cautioned that high parental expectations may cause learning to become too regimented, which may frustrate children and discourage their future engagement:

“*Some parents want their child to be the best, don’t they. They have a vision of them reaching a certain standard. They sometimes push too hard with unreasonable expectations and then get frustrated when they can’t do it. It can put them off. I saw it with my husband who is really into cycling and was really pushing down the skills route with it. So, maybe expectations can take over, and we need to find that balance with teaching skills as parents.*”

(TE3)

### 3.6. Rules and Competing Demands

#### 3.6.1. Risk Aversion

Participants discussed risk aversion, with many noting that while children may occasionally get hurt, allowing them to take controlled risks was necessary for FMS development:

“*To a certain extent you’ve got to let them take those risks. There is always a chance that they are going to get hurt. My eldest broke his wrist on the second day of school playing on the adventure playground. But I think the benefits far outweigh the risks.*”

(PT5)

“*At some point you have to step away and give them that freedom to have a go without you being stood underneath ready to catch them… It is important that they learn how to problem solve and how to get themselves down safely once they have climbed up on something.*”

(FP2)

But there was shared concern that well-intentioned restrictions were being implemented by caregivers to prevent injury, and were negatively impacting children’s FMS development:

“*There are a lot of families that say, ‘he climbs on everything’ and are like ‘he can’t climb up there, he will fall.’ There is such worry from parents nowadays about children being injured. But his body is telling him to climb. Just stand by him and let him develop his skills. I don’t think there is enough understanding from parents that this is a natural instinct within a child.*”

(FP1)

“*One child who wasn’t allowed to access the frames at school because they’d fallen off previously and cut their head. So, the parents said they wouldn’t be going on again, even after weighing up the benefits… This was one of those accidents that couldn’t have been prevented.*”

(TA1)

“*I was at the park with my two and another parent wasn’t letting their child on some of the things in case they fell. It’s natural risk aversion and that bubble wrapping has been instrumental in the downturn of children’s skills development.*”

(PT3)

Some participants believed that caregiver anxieties could limit children’s active play opportunities, particularly grandparents’ fear of being blamed by parents for any injuries:

“*I am more careful with my grandchildren. Say if they were in a play area, I am very much more, ‘oh, be careful, oh, don’t do that’ compared to how I was with my own children. Because they are your responsibility, and they are not your children. If they hurt themselves, the parents are going to ask what we were doing. You would get the blame for it. You would, it’s true.*”

(GP2)

“*It’s sometimes because parents think somebody might say something about the bruises on their child. Especially the families with children who might be going under the radar.*”

(NW1)

“*Looking back at my own experiences as a child, my dad would let me do whatever I wanted at the park. Whereas my mum would stop me from climbing trees. I do feel it differs stereotypically between boys and girls. The other week I saw a parent with their children, and the son was climbing, and the daughter went to copy him. But she was the only one told to get down.*”

(PS1)

One family hub practitioner felt that wider societal fear of risk was influencing practice, including play space design, and limiting children’s opportunities to develop physical skills:

“*Years ago, risk was allowed. In the whole of today’s society, there is this sense of keeping children safe and trying to make playgrounds safer. But by making them safer, we are taking away imagination and risk. We are stopping children from learning their own boundaries and it is putting limits on physical development as well.*”

(FP1)

#### 3.6.2. Caregiver-Mediated Outdoor Restrictions

Several participants valued children spending time outdoors, even seeing adverse weather as fun and choosing to play outside despite unfavourable conditions:

“*It’s rare we stay in all day. If we have a day when we aren’t doing anything, then they’ll want to get out on their scooters or bikes. Even in this weather, when it isn’t warm and sunny outside.*”

(PT1)

“*Yes* [likes to play in the rain]. *But only if I put my coat on so I don’t get wet and cold.*”

(CH2)

In contrast, there were many examples where caregivers found outdoor activities less appealing in the winter months and would purposively avoid taking children out to play if it was wet or cold:

“*It does put us off in the winter. In the summer we are a lot more eager to get them outside and to parks and stuff. But in the winter, it’s colder and it’s wetter.*”

(PT2)

“*I personally hate being wet and cold. If it’s raining or snowing, or if it’s anything below fifteen degrees and my kids ask to go out then I’m like ‘not today, it’s freezing.’ Warm weather makes it easier, and we are without doubt far more active in the summer than in the colder months.*”

(PT5)

“*We don’t always want to go out and play or go to the park at this time of year. The weather is a bit rubbish, and it gets dark really early as well. You can’t just go out and do things.*”

(PT7)

Caregiver avoidance of poor weather was also reflected in children’s perspectives, who implied that caregivers had set seasonal or weather-related boundaries on outdoor play:

“*Only in the summer [playing outside]. Because it might be too cold and wet in winter.*”

(CH2)

“*We have quit football now. It is getting colder. Mum says it’s too cold.*”

(CH7)

Educators described situations where caregivers would try to restrict children’s outdoor play in bad weather to avoid illness, which was compounded by children being inappropriately dressed:

“*Parents don’t seem to think you can go outside when it’s raining. We get parents saying not to let them outside because they might catch a cold… Some little girls will come into nursery with flipflops and fancy dresses on… They can’t join in and play in the mud. Or the kids will come in the middle of winter with a flimsy coat on. They aren’t prepared for outdoor play.*”

(NW1)

“*I’ve found through my work, as a family visitor, a nursery nurse, and a Scout leader, that many families are outdoors adverse. So, it’s about having discussions and explaining that rain doesn’t mean you can’t go out. Put a coat and some wellies on and provide those experiences like jumping in puddles. There’s no such thing as inappropriate weather, it’s inappropriate clothing.*”

(FP2)

Additionally, participants recalled caregivers restraining children in pushchairs or carrying them when they were capable of walking, reducing their opportunities to develop mobility and FMS:

“*I do wonder how much children are being carried in this age group. We have a year one child whose mum still picks them up and sticks a dummy in when they come out of school. I see pushchairs being used for far too long too and that is why some of them can’t walk properly or don’t have that core stability or balance.*”

(TE2)

“*Lots of them come into school in pushchairs. They aren’t walking. They are pushed everywhere. I think it’s convenient… You’ve got all these bags to carry and no hands free to hold your children’s hands. Whereas if they are in a pushchair, they’re not running away, and you have somewhere to store all your things. It’s an easier life for you, but it isn’t for the benefit of the children.*”

(TA1)

One practitioner explained how caregivers could set clear boundaries for young children, which would allow them to move with more freedom without compromising their safety:

“*Some of the families I work with are like, ‘the children won’t hold my hand. They’re running off.’ So, let’s set them some boundaries. It’s safe to let go of your hand and run within reason. Just set the limitations.*”

(FP1)

#### 3.6.3. Household Rules

When outdoor access was limited, both children and caregivers described indoor active play at home as enjoyable and supportive of FMS development:

“*I like to play The Floor Is Lava inside.*”

(CH7)

“*We do a lot of wrestling and play fighting. If we are having a day indoors, we like to get things out and build dens or play games like The Floor Is Lava or Hide and Seek. So yeah, they are still being active running around the house all the time basically.*”

(PT5)

“*Another game we play is The Floor Is Lava. We bought some cheap circles that we scatter on the floor in the house. They love that one and you can play it pretty much anywhere, hopping and jumping around and interacting… We encourage it* [indoor play] *because I think any child would love making sofa forts or dens under the table, and things like that.*”

(PT9)

However, some caregivers enforced strict household rules to prevent injuries or damage to the home, which constrained children’s active play and FMS-building opportunities:

“*My daughter was forward rolling between the bedrooms, and my husband comes in shouting ‘don’t forward roll there.’ But I was like ‘leave her alone.’ As long as she isn’t about to fall down the stairs or injure herself. My bug bear is that he is telling her to be careful all the time indoors.*”

(PT3)

“*They do just naturally want to pick things up and throw them. But we don’t let them play with the ball or chuck things around in the house so that they don’t knacker everything up.*”

(PT2)

“*Some parents have the no throwing in the house rules. But if you can’t go outside and do that at that time, then how do they burn off that energy and practice those skills?*”

(FP2)

Indoor rules also featured heavily in children’s interviews, with some being particularly expressive as if they had been conditioned to see indoor PA as unsafe or wrong:

“*No running inside… Things will break.*”

(CH1)

“*Not running. Just walking… We might bump ourselves into something.*”

(CH2)

“*She says stop. Stop running inside because I might crash, and I might hurt myself.*”

(CH9)

“*My mummy and daddy do let me play games in the house, but they don’t let me run around… I might break something, and sometimes they mop, or I might fall down the stairs. You would go whoosh! Then you would have to go to the doctors, and you would have a bandage on your head, and you would have to have a rest in your bed.*”

(CH10)

Both family hub practitioners were familiar with the issue and recalled conversations with families about how to appropriately support children’s indoor PA rather than discouraging it:

“*We have discussions with families who say they have issues because their child likes to throw things. But it is not an issue because the child is learning by throwing things. Rather than throwing toy cars and hitting the TV, we can get some balled up socks to throw that won’t cause damage. That way we are supporting them, and they are having fun even though they are inside.*”

(FP2)

“*I suggested that we could just move that coffee table out the way. That way you are not going to bang yourself on it while we hop across the room or something.*”

(FP1)

This practitioner advice aligned with the viewpoint of one of the caregivers, who expressed a flexible and tolerant approach to indoor play that supported their child’s movement practice:

“*You can practice their movement skills anywhere. It doesn’t need to be just outside. You can’t really ride a bike inside, although she does love to try. As long as what she is doing is safe or what she is throwing is soft so it won’t break owt, then course she can play inside.*”

(PT6)

#### 3.6.4. Workload and Time Pressures

Time poverty and the challenge of balancing work with family commitments was widely recognised as a major barrier to supporting children’s FMS, particularly if both parents worked:

“*A lot of parents work and don’t finish until at least 5 p.m. If they finish at 5 p.m. then where is the time to be physically active with your kids or to take them to clubs?*”

(PT4)

“*Time is the biggest issue we have. We are both full-time working parents, so life is just a constant juggle. I think quite hectic would be putting it mildly, and work doesn’t equal school hours. If work was 9 to 3 then we would be laughing. We’d have loads more time to do things.*”

(PT8)

Participants discussed how varying work schedules, long hours, and blurred boundaries from remote work further reduced time for play and engagement with children’s FMS:

“*With my previous work, I was at work before the children started school so they would go to a breakfast club. It was always a rush. I would work really long hours, pick them up after 6 p.m., and it would almost be my son’s bedtime. Home, bath, bed. Life was busy and add in shopping, cleaning, and organising, there was just no time to be active with the children.*”

(TA1)

“*I worked nights for many years, and I didn’t see my family as much because I was at work in the evenings. I can see why it would be very difficult to do this [work on FMS] on certain days, because it’s got to fit around parents working.*”

(GP4)

“*Many parents work so much now compared to twenty or thirty years ago, where maybe one parent could afford to stay at home and play with the children. I think a lot more parents are working from home now too, which involves a lot more of ‘please sit down and be quiet. Mummy is working, daddy is working.’ It has brought the workplace into the home and now there is less of ‘oh, daddy’s home from work and is here to play’ now. This has gotten lost in recent years.*”

(PT5)

Parents reported post work fatigue limiting their capacity to support children’s FMS, which would occasionally lead to stress and family tension if they attempted to engage:

“*If you are working sixty hours a week, as harsh as it sounds, the last thing you want to do when you come home is to start running about having a mad one with your kid. It would be down time, have a bath, and get something to eat and drink.*”

(PT6)

“*She goes to school full time, and we both go to work full time. By the time we are all home, we are all absolutely knackered, and playing and doing physical things are the last thing that we want to do as a family. We all get ratty with each other and argue.*”

(PT7)

Several participants highlighted the negative impact of parental work commitments on children’s PA opportunities. One child poignantly drew their whole family at the park despite their father being absent in reality, reflecting their frustration at not being able to play together:

“*We haven’t got enough time because I’m always at work. There is a dance group near us that she went to at some point this year. But it wasn’t appropriate with my work timings. She needed to be there by 4 p.m. and I was never getting home in time.*”

(PT6)

“*There was one boy who I felt had really good skills and brilliant hand eye coordination, and I said to his mum, ‘have you ever thought about going to All Stars Cricket?’ But they said they were working and hadn’t got time to get him there. It was such a shame.*”

(TE2)

“[On why their parents cannot always play with them] *I don’t know. Because they are busy. They are always busy. I tell them to play but, well, they don’t play. They are at work… I went to the park yesterday, but without my daddy. But everybody went to the park in my picture.*”

(CH8)

Family hub practitioners countered that even small amounts of time, planned carefully, can provide children with vital opportunities to improve their FMS:

“*Whether parents are working or not, there is always that little bit that they can do. Half an hour or even 15 min each day. Maybe an hour or whatever at the weekend when there might be a little bit more time. It will just give the children more time to perfect those skills.*”

(FP1)

“*One of the barriers that parents communicate to us is lack of time, but you can plan that time better. We are then looking at time management and seeing how we can do it. We also have lots of parents that say they have housework to do. But that can wait. You could easily spend that time being active with your child instead and when they have gone to bed you can do it then.*”

(FP2)

Grandparents argued that they had more time and flexibility to support their grandchildren’s development compared to parents, which was echoed by an educator:

“*Grandparents are often no longer working. So, when the grandchildren come here, the day is dedicated to them. I don’t do any cleaning on those days. Whereas if you are a parent, you have other things that you have to do, and if you don’t do those things, you will have no food or clean clothes. But as a grandparent it’s different… You have more time for them.*”

(GP2)

“*Have they got enough time in the day to do these things because of work. But this is nothing that can’t be resolved between the parents and the grandparents.*”

(GP4)

“*I think it is the grandparents more often than not that are taking them to the park. Grandparents are often picking up from school. You see loads of kids with grandparents walking around and going to the park. Because often the parents are obviously at work.*”

(TE2)

#### 3.6.5. Academic Pressures

Participants discussed how some parents would prioritise their children’s education over physical development:

“*I don’t think they [FMS] are a priority because they’ll just come along later on. I think other things take priority, like education. Education is important because that is something you can work on immediately. Unless you want them to be an athlete, you need to focus elsewhere.*”

(PT6)

“*There are some parents that think a certain way. They want them to write their names, and they think they are going to be doing maths. You will get some children at nursery who can tell you the alphabet already. It’s repetitive and correct, but they don’t actually know what it means. Well, they don’t need to be doing these things this early. They are here to learn how to play.*”

(NW1)

Some participants felt that parental emphasis on education was often driven by cultural expectations, where success was defined more by academic success than physical ability:

“*Within certain cultures other things take precedence. Some cultures focus very much on academia, and so it’s homework, it’s tutoring, it’s reading. It’s not because they don’t want to do physical activity, it’s just in that culture or as a family that’s what is prioritised.*”

(PT4)

“*Different cultures have different beliefs. There are cultures where part of their cultural system is academic attainment. Education, education, education, extra tuition, playing an instrument, you are going to be a doctor… The idea of just going out and playing for pure enjoyment doesn’t even come into it. Going to the park or having a kick around, it just disappears.*”

(FP2)

An opposing viewpoint was that academic pressures stemmed from the education system, with early formal schooling and homework reducing families’ time to support children’s FMS:

“*Our children go to school much earlier than other European countries and are forced to sit still and be educated rather than being allowed to be active and develop physically. There is so much emphasis on academics… This focus on academia is leading to a lot of time pressures on parents who maybe only have a weekend to do activities… Such a short window of time.*”

(PT1)

“*Schools put a lot of emphasis on homework. Reading, writing, and spelling… They expect you to do this so many times a week. This cuts into the time you have to do other stuff… I understand the importance of homework, but I do think parents know better sometimes on whether children should be doing homework or spending more time on physical activities.*”

(PT9)

Educators acknowledged that schools sometimes focus more on core subjects than on PE and FMS, and would provide less communication to parents about PE than about academic tasks:

“*For example, when we do parents evenings we have a script. Let’s talk English and maths. Let’s talk attendance, behaviour. We are not talking about anything else, really. So, we are totally missing the physical side and there is a massive gap there. We are supposed to be holistically educating children, not just how to read and write.*”

(TE1)

“*I’ve had a battle at school that has upset me. I am English lead, and I have said not to hear readers during PE. But they are like ‘well, we never get to hear them read otherwise.’ But those children need that PE lesson too.*”

(TE2)

One parent believed that academic pressures not only limited families’ time to focus on FMS development, but also left children too tired to engage in activities at home:

“*I do think a lot of pressure is put on children these days in terms of all the schoolwork. They are worn out from all of that before they can do anything physically. When they come home in the evenings, they don’t have the energy left to go and do these activities.*”

(PT9)

“*When you pick them up from school, they might not feel like doing anything else. They are often too tired and just want to chill out.*”

(PT2)

### 3.7. Lived Environments and Life Circumstances

#### 3.7.1. Outdoor Spaces and Active Play Environments

Children spoke fondly about their gardens and the features within them, while adults emphasised how they support activity and skill development, and created cherished family moments:

“*That’s me. That’s my trampoline. That’s a rainbow and some grass. It’s at my house, outside in my garden. It’s got two hills. One goes down, and one goes even downer to our flower meadow… We do races. It’s fun. I can run around and roll and go up and down and up.*”

(CH2)

“*I would play my game in my garden. I would probably hide in my tree house. Or I would hide in the trampoline.*”

(CH10)

“*In the summer especially, the garden becomes an extension of your house and becomes an extra room for activity. This is the reason why we built all the stuff in the garden. It’s for all of us to play on. We have got a trampoline, and we have a tree house that has swings on it and a climbing section… It’s all about making memories that I cherish and hopefully they do too.*”

(PT9)

“*I was really lucky when I was growing up that we had a really big garden. I would practice handstands, cartwheels, and we always had the paddling pool out. I had lots of equipment like skipping ropes, like playing on bikes and basketball hoops and all these things.*”

(TE3)

Families similarly appreciated community parks and green spaces, seeing them as important for active play, adventure, and connection with nature:

“*The park is just a stone’s throw away so we quite often go there. We might pop there after school and the girls are very happy there. It is in a beautiful setting as well and right by some open fields. It’s nice and safe and has a nice outlook and you have access to the great outdoors around you. I grew up in a lovely village myself and that was a big priority for me and why we decided to move to where we are now for that community feel.*”

(PT11)

“*We go out for lots of walks. He loves running about on the grass on the fields and playing on the park with all the climbing frames and slides.*”

(GP4)

“*We go to the park. On the big girl swing.*”

(CH3)

“*Sometimes we play at the park near our house.*”

(CH7)

Crucially, family hub practitioners emphasised how outdoor spaces provided children with important opportunities to develop their FMS:

“*There are a lot of lovely open spaces across Derby for them. You’ve got Alvaston Park, Elvaston Castle, and Sinfin Park that we go to that are just lovely environments to get children balancing, running, jumping, kicking a ball. They are full on positive aren’t they.*”

(FP1)

“*Climbing frames in parks give them the opportunity to build upper body strength and develop coordination to go up an object and then come down again. To get to different levels to jump so that they can learn to negotiate and land safely as well… One of the groups I have been running recently is around connecting with nature, focusing on the importance of being on grass or in wooded areas to support movements, and being able to negotiate different terrains rather than just using smooth paths with sure footing… Parks and green spaces help support development a lot more than having an open area with some Astro turf.*”

(FP2)

Caregivers and children also spoke about informal, movement-rich extracurricular settings as being enjoyable and stimulating environments that supported PA and FMS practice:

“*We have taken them to entertainment centres where the kids would bounce and go in ball pits. Now they like to go climbing at Clip and Climb. They are brilliant ideas whoever came up with these concepts, because it encourages movement, and it develops their abilities.*”

(GP1)

“*Playgroups are a good environment. They are playing and meeting other children and doing all those movements from an early stage in life. We also go to the swimming baths with him. It’s a different environment and different movements, but he’s still learning.*”

(GP4)

“*I like the trampoline park because I can run around and they have a really big slide.*”

(CH5)

“*I like swimming… It’s more fun going to other places.*”

(CH1)

Caregivers additionally described sending their children to structured extracurricular activities and clubs to further support their physical, social, and psychological development:

“*She does a lot of additional activities, just because I think it’s important. She does football, horse riding, taekwondo, and she swims. Each activity brings something different… I want to provide my children with what they need to succeed, and that includes improving their physical ability.*”

(PT1)

“*She goes dancing twice a week, she goes to swimming lessons, and she goes to rainbows where they are playing a lot of games and running around. I think it helps keep their minds and bodies healthy, because you can run out of things to do at home sometimes. So, these activities get you out of the house and moving and doing something healthy.*”

(PT5)

#### 3.7.2. Structural Inequalities and Physical Access

Although outdoor spaces and community amenities were central to family engagement with FMS, physical barriers and the loss of local facilities restricted children’s safe practice opportunities:

“*In my old house our garden was on quite a slope. The space wasn’t there even though we did have a garden, just because of the orientation of the land. So, I can only imagine what it would be like for people who don’t have a garden.*”

(PT4)

“*Three of the parks I used to go to when I was a kid have closed down. One is just a grassy area now, one has become a car park, and one is just a mud pit. All those parks have gone.*”

(TA1)

Some participants argued that access to local spaces depended on geographical location, and sometimes assumed that there would be fewer outdoor opportunities in more populated areas:

“*I think we are in a really good area to be honest. We have something like five parks within walking distance, and it’s nice to be able to mix it up and go to different ones and do different things. I think we are lucky compared to other areas that don’t have as much around them.*”

(PT10)

“*I feel like we have got plenty of space and general parks around us. I would say we have got enough within walking distance of us. We are lucky in that respect. But if you were in the inner city, you might not have that green space around you.*”

(PT2)

Whereas one grandparent explained how living in populated areas does not necessarily limit access to parks, arguing that London offered more opportunities for children than the north:

“*It surprised me when my daughter first moved down to London how many parks there were all around. There is a hell of a lot of green areas where kids can play. I would even argue there are a lot more places closer to hand and easier to get to than up north in Wigan. It’s weird.*”

(GP4)

Some participants questioned whether parks and open spaces adequately support all children’s FMS development, noting that designs often target toddlers and lack appropriate challenges:

“*Parks in new estates are very much geared around toddlers. They need more advanced things, like the spider’s web climbing nets… Or think of a school playground with snakes and ladders or grids painted on the floor that they can jump on. You don’t always get this in local parks.*”

(PT8)

“*My children are interested in basketball. But I don’t remember seeing any local facilities with basketball hoops. So, unless you have a hoop at home, you are not going to be able to practice this type of skill where we are currently.*”

(PT9)

Another determinant that reportedly influenced outdoor access was road infrastructure and traffic, though several caregivers still made intentional choices to model active routines:

“*I always make sure we walk on the school runs. Like, if we can walk to the shops instead of driving, then we will aim to walk there as well.*”

(PT4)

“*I ensure that we all walk to school. I’m quite keen to instil that and it is a good way to get half an hour’s walking in a day there and back. We do that come rain or shine.*”

(PT8)

Additionally, several parents explained that quiet, low-traffic locations around the home improved feelings of safety and encouraged children’s engagement with activities:

“*She likes to ride her bike and scooter on the front. Thankfully we live on a Cul de sac and we have a private bit where there aren’t many cars running about.*”

(PT7)

“*My daughter loves to go out on her bike. She doesn’t even need to go far because we have got a nice area at the back of our house that is flat and there are barely any cars. So, she will just spend an hour literally going up and down out the back. She loves doing that.*”

(PT10)

However, other caregivers were hesitant about supporting active travel or allowing their children to play in outdoor spaces around their home and community due to road-related dangers:

“*We go on our scooters to school. But it is a busy road and sometimes my heart is in my mouth because there are big lorries and cars steaming by. Somebody else said to me they wouldn’t dare take their children to school on their scooter because it’s not safe… Schools keep promoting active travel but if parents don’t feel safe nothing will change.*”

(PT3)

“*We live in a village with a huge main road cutting through it in front of our house. It’s supposed to be a 30 mph limit, but it very much isn’t. There are a couple of crossing points, but there’s nothing to help you get across to the park. We’ve had a couple of really scary encounters where there have been massive lorries speeding through, and my daughter has nearly gone out into the road. We wouldn’t be able to let the children go out on the front to play.*”

(PT11)

To improve safety for active travel, one educator described how their school had implemented enforced local road closures, but this had unfortunately displaced traffic into surrounding areas:

“*Road closures around schools during key periods is a good thing. The area is quieter and feels safer for families to take part in active transport. But it can push the problem elsewhere. People just park on the next closest street. There are too many people in too many cars, all rushing to drop off and get to work. I don’t know how we reverse that behaviour, because if we feel the roads are dangerous for walking or cycling, we choose safety over health and fitness.*”

(TE3)

#### 3.7.3. Social Influences and Family Circumstances

Families faced several socioeconomic and environmental barriers that limited children’s PA and FMS development. Neighbourhood safety was a key concern, sometimes extending to the home:

“*It is quite a deprived area, so you tend to find that some areas are just not safe. There are needles in the parks and stuff has been vandalized. So, whilst there can be a lot of good things in parks, there is that element of safety, and would you actually use the parks is the question.*”

(PT4)

“*In places where we lived previously you wouldn’t take your kids to the local park. You didn’t feel safe there. There will be dodgy dogs on the loose, and needles and broken glass. I’d rather keep them inside rather than exposing them to something that might harm them.*”

(PT5)

“*We do actually have dogs, so I need to be careful because mummy doesn’t want me to step in anything bad that they have done.*”

(CH10)

In addition to safety concerns, parents felt the cost of both structured and informal activities were increasingly unaffordable for families, creating financial barriers to children’s movement:

“*My daughter really enjoys gymnastics, but it is eighty pounds a month to go. So, as much as she would enjoy it and benefit from that, that’s not affordable.*”

(PT5)

“*Certain activities are not affordable for families. Oxygen* [Indoor play centre] *for an hour for two children is over £30. That’s a lot of money for just an hour. We are lucky that we can afford these things, but I wouldn’t say it is affordable for less fortunate families to be honest.*”

(PT10)

Furthermore, access to transportation and travel expenses were also viewed as barriers to children’s participation in activities, especially if these opportunities were beyond the local area:

“*My sister can’t drive and she struggles to get her kids to activities unless it’s within walking distance. So, it massively impacts what activities her kids can do outside of school.*”

(PT2)

“*If there’s nothing locally, it might be a long way to travel if you don’t have a car. You could use public transport, but if you get a bus it takes time, and all the difficulties involved in that. I know with the buses the government introduced a £2 price cap, but they just stuck that up to £3. So, if you have multiple people that’s mounting up.*”

(PS1)

Participants detailed how these pressures were greater for families experiencing financial hardship, whose children would receive less opportunities to practice their FMS:

“*I had one family that we had known since the children were 4 and 5 years old, and they came back on our radar aged 11. After all that time, the mum and two children had been living in a one-bedroom flat. No garden. The three of them were on top of each other.*”

(FP2)

“*I have been to households where they are struggling financially, and they just don’t have any toys or equipment, and the children’s motor development is suffering… They have other priorities like heating your house or food.*”

(FP1)

“*Some families are in one room flats with water running down the walls. Those families wouldn’t be able to afford extra activities because it’s all pay this and pay that. Parents can’t always afford it and so the child will be excluded.*”

(GP1)

Educators outlined how socioeconomic pressures often translate into differences in children’s physical development, with experiences and FMS varying by affluence or area deprivation:

“*I did a* [work] *placement, and I was doing that in the mornings and then working* [their usual job] *in the afternoons. It’s only one long road between, but the difference is huge. They could all ride a bike in Chellaston, but at ours they couldn’t. It’s the area, isn’t it.*”

(NW1)

“*It depends where the children are demographically. We took a class of 30 swimming last week for the first time. Only four of them had been swimming before and could swim.*”

(TE1)

Nevertheless, many participants challenged the need for resources for FMS development, emphasising that families can creatively repurpose everyday items to support movement:

“*It might be as simple as putting cushions on the floor and getting them jumping over them. What household doesn’t have something soft that can be thrown or caught, or something that can be used as a basket. It’s not about expensive equipment. It’s about being imaginative with what you have got and being comfortable with that.*”

(PT3)

“*You can do this with little or no equipment or cost. Some families would bend over backwards to get their child into a football club or buy all this equipment, and some just can’t afford to do anything. So, what can we use around the house? Balled up socks cost nothing. Everyone will have a basket or saucepan* [to throw into]*, so you have got what you need there.*”

(FP2)

Participants also identified school-based initiatives that funded inclusive participation and helped reduce financial barriers for those children needing extra support:

“*We have money set aside for pupil premium children. We identify children who need extra support in their physical development and if the parents are struggling for money, they’ll get a free place. Because we are aware some of those children don’t get everything they need.*”

(TE2)

“*At the school my daughter goes to, they do after school clubs and activities. They are usually free or a couple of pounds per session. Things like that are really helpful. It would be nice if more of this was made available because for a lot of parents the money is the barrier.*”

(PT5)

But one parent felt that providing subsidies only to the most deprived families can leave working families struggling and increase inequality for those children who do not qualify:

“*I do think disadvantaged families get a lot for free. Extra stuff like the holiday clubs, which are so expensive to working families. I don’t always feel it is fair, but I accept it. I think two full time working parents just get screwed across every aspect. I feel like we get penalised and what I don’t think is fair is the impact on the children.*”

(PT8)

#### 3.7.4. Family Screen Time Behaviours

Participants felt that screen use had escalated in recent times and were concerned that children were becoming less active and spending more time on sedentary, screen-based activities:

“*I think it has become a general societal issue. Children are on phones and tablets or sitting still watching telly. Children are not in play areas running, hopping, jumping, or interacting, and that natural exploration is not happening. But their bodies weren’t made to sit still. Their bodies were made to move and wriggle right from the word go.*”

(FP1)

“*I didn’t grow up with a phone or an iPad. We had the TV for an hour after school sometimes. But ultimately it was going outside and playing stupid games until bedtime. That has changed now with all this technology, and I think children would be a lot more active without it.*”

(PT5)

Parents recognised that screen time was increasingly used to soothe or occupy children, and that caregivers’ own device use often directly influenced children’s screen-based behaviours:

“*Too much screen time is not great for any child. But for adults it is convenient so you can get on and make the dinner and get ready for the next day. It’s awful to say it out loud but it is just an easy thing to sit them and distract them so you can get on and do things.*”

(PT7)

“*I’ve seen it where the child has cried, and the parents have just given them a phone to calm them down instead of talking to them or doing anything with them. It winds me up. I just wonder in 20 years’ time are any of these kids going to be talking or doing anything with each other.*”

(NW1)

“*The children are seeing and copying what their parents are doing. I’ve seen it in restaurants where parents are on their phones and the children are on their tablets. One of the children knocked their drink over and the parents didn’t even notice. You think to yourself ‘get off the damned phone. Get off that phone.’*”

(GP1)

Educators believed that excessive screen use was impairing children’s imagination, social skills, and their connection with nature, and linked unhealthy screen habits to poor motor competence:

“*Children are sat more often at home on tablets and devices, and because of this I have seen a lot of children with very poor core ability and are struggling to sit for periods of time. We have also got a lot of children who are poor in the way they move their bodies. Some can’t jump yet.*”

(TA1)

“*Children are generally sat on screens… They’re not naturally developing their gross motor skills because they are not in the trees or in the mud outside… They don’t know how to play anymore because screen time is either limiting their imagination, or they are mimicking computer games and play fighting, which leads to actual fighting sometimes when it escalates.*”

(TE1)

“*Children who are on screens constantly, their imagination has been taken away. So, when you put them in a field to let them run wild, they struggle. They don’t know how to play or what to do.*”

(FP1)

Educators highlighted how screen use can affect children’s wellbeing, including sleep, concentration, language, and behaviour, based on both professional and personal observations:

“*I used to use tech as a babysitter a lot myself. But we noticed my son wasn’t sleeping well, and we went through a sleep consultant who said scrap the tech because of the blue lights. We then started stricter TV times and he’s never been happier.*”

(TA1)

“*Thinking of my own experiences with my daughter, she has to be given a countdown to end the screen time. Otherwise, if you just cut her off, she can’t regulate it and will have a bit of a meltdown… They are so absorbed by it that you sort of have to wean them off.*”

(TE3)

“*We’re having issues with language at the moment. It’s very American and they are clearly picking things up from YouTube. Focus is appalling. There is one child who is hitting and kicking people and using the ‘C’ word… Grand Theft Auto springs to mind because he has a gaming unit and a gaming chair and everything, and he is only five.*”

(TE2)

A practitioner explained how caregivers were being left uninformed about healthy limits and the risks of excessive screen exposure due to a lack of guidance for caregivers to follow:

“*Technology is such an easy answer and there are no guidelines for screen time hours. There isn’t anything concrete to follow. Schools are using it now too. Then they come home and have more screen time. Guidance is really needed on how many hours and on whether it is for educational or entertainment purposes.*”

(FP2)

Despite extensive discussions on screen time, no children drew technology. However, one 3-year-old spontaneously recalled the influence of TV and computer games on their play after noticing a Telly Tubby figure:

“*Look, Dipsy has a TV screen on his tummy… I watch TV…. I like to watch Hot Wheels…. I like to copy the cars. Dumpster is stinky… I play with pickaxes like in Minecraft.*”

(CH9)

The relative absence of child testimonies on screen time reflected adult views that screens have become so normalised in children’s lives that they no longer associate them with play:

“*I don’t think children see it as play anymore. They just see it as something to do. There isn’t any interaction, and they aren’t drawing anything from it. I think screens would be the last thing on their lists of what they liked to play because I think they have normalised that behaviour.*”

(FP1)

“*I don’t think children are seeing screen time as play. I think it’s just the norm and it’s just what society is now. I don’t think it’s a case of ‘oh, let’s go play a game on your iPad.’ I think it’s more ‘here’s your iPad,’ and that is the difference between the two.*”

(TE1)

However, not all participants viewed technology negatively, with several highlighting how screen-based activities and online resources can support children’s learning, skill development, and home participation:

“*You can use screen time to your advantage. Switch Sports. They love doing physical activities using that. You can put YouTube videos on and use things like Just Dance for them to dance along to and work on their skills that way.*”

(PT9)

“*You can just pop YouTube on and dance in the living room. It can be used positively. It’s a bit like alcohol though isn’t it. In moderation. Because you will lose the benefit of it if you overuse it. But if a parent can’t afford to send their child to an activity, it doesn’t have to stop them because they can use the TV. They are just learning in a different way.*”

(PT6)

“*During the COVID lockdown I used to send videos of exercises to parents to support their children’s skills at home. The feedback was really good and in fact they were like ‘we’ve been practicing and now as a family we are all joining in.’ We had Joe Wicks at the time, didn’t we. It was those little nuggets of information sent in this way that parents liked.*”

(TE2)

### 3.8. Relationships

#### 3.8.1. Family Ethos: Foundations of Engagement

Participants contemplated what family meant to them personally. The caregiver-child relationship was considered central to children’s lives and key to their support and development:

“*Family is very important to me. It’s mine and my husband’s responsibility to raise these children who are the next generation, and, in that sense, it’s a big responsibility being a parent. We are teaching them, they are learning from us, and we are the ones our children rely on. I heard a quote the other day that said, ‘You are giving your child a childhood that they don’t have to recover from.’*”

(PT4)

“*Family is the most important thing. To have a family is something that we have always wanted, and our number one priority now is for our children to have a good family life and nice adventures.*”

(PT8)

“*To me, family is everything. I see my family as role models, and I am living my life with the morals and the values that they taught me.*”

(TE1)

Educators connected family directly to FMS, describing the home as a setting where opportunities were enabled or restricted by caregivers as gatekeepers to children’s participation:

“*Families are really important. Because the whole culture and ethos of this is embedded in the family home. How physical a child is can impact on the rest of their development. So, if they are not interested in it then that’s quite detrimental to the child. That makes it really, really important then that families are aware and involved in this.*”

(FP1)

“*Families are the most important, out of everyone. They have that influence. If they are not taking their children to parks and clubs, or if they don’t have that basic understanding, then nothing is ever going to come of it. You need that parent to be involved or take you to the park or club. Not the teacher. So, they are the most important.*”

(PS1)

One educator suggested that PA itself provided families with quality time together that strengthened familial bonding:

“*I think it is very important spending time with your family. I think sport, and play, and activity can help bring you together as a family. It is quality time, so yeah, I think family is really important.*”

(TE2)

#### 3.8.2. Social Interactions: Peer and Sibling Influences

Children highlighted the importance of socialisation, enjoyment, and a sense of belonging. Adults discussed how friendships and shared activities support confidence and personal growth:

“*I have play dates with my friends… They go to school with me. They are at the same school and in the same class.*”

(CH2)

“*That’s my friend called Lucy* [in the drawing].”

(CH11)

“*I can run around with the children at the trampoline park.*”

(CH5)

“*She has made friends through her dance clubs and Squirrels group. I think this helps to make them feel more confident, and when they come up against some challenges they might think, ‘well I have tackled this before, and I believe I can do this.’*”

(PT9)

“*I went to my friends the other day and all our grandkids were together. We went to the woods, and they all started making a den together, dragging logs along and everything else, working together on it.*”

(FP1)

Some participants recognised that socialisation is important for children’s FMS development, with peers providing the stimulus to practice and refine skills, and schools acting as key settings:

“*I think children learn through other children… Copying what other children do. Otherwise, they would just stagnate. They need to socialise to improve in their movements.*”

(GP4)

“*I do think it is environmental at this age. Ever since she’s been going to school, she’s picked up loads that I don’t think she’d have done if she hadn’t been at school with other children.*”

(PT7)

Children similarly recalled positive play with siblings, and caregivers agreed that brothers and sisters enhance engagement in both casual active play and structured FMS practice:

“*Because my sister chases me* [why they liked playing together]. *She shares everything with me. We get to play funner games.*”

(CH2)

“*I like playing with my sister because then we are silly. It is more fun.*”

(CH5)

“*I play with my brothers. Wresting, and we arm wrestle.*”

(CH9)

“*Oh 1000%* [multiple children can facilitate engagement]. *I was even thinking today, ‘wow’, because there are four of them, they can actually play a game of football two on two. So, they have those extra opportunities to play.*”

(PT4)

“*I think they play more because they are doing it together. It’s better when you are doing it with somebody else. I think if you were on your own, it would become boring. So, having brothers and sisters, yeah, I think it encourages you to take part.*”

(GP2)

Parents believed having multiple siblings supported independent play and reduced the need for caregiver involvement, with older siblings acting as role models for skill development:

“*I had my children all quite close together. So, I would say there is less involvement from my side* [in FMS development] *because of this, but she has her siblings to learn from, so that helps her. Having someone to copy, follow, chase. It even helped her ride a bike, and things like that. She has seen her older brothers do it and wants to be part of that club.*”

(PT8)

“*My daughter was a lot quicker at picking up skills than her older brother. I think this was because she had got him there. He was running around so she wanted to run around. Therefore, she walked quicker than he did. She wanted to play football with him, so she was kicking younger than he was able to. She did it all quicker because she wanted to be like him and play with him.*”

(PT10)

On the other hand, participants detailed how siblings could disrupt activities, which would cause arguments and bring play to an abrupt end:

“*I know watching my two, there are times when they play together lovely, but also times when they just fight.*”

(TA1)

“*My three- and six-year-olds were playing in the garden on their own when they started fighting and one of them fell out of the tree house.*”

(PT3)

“*Sometimes my sister plays with me but sometimes she isn’t very nice to me.*”

(CH10)

One parent shared how their eldest child’s serious illness had negatively influenced their youngest child’s FMS development, as the younger child began mimicking the restrictions:

“*She was definitely influenced at the time of his illness. He spent a lot of time at the hospital. He wasn’t interested in moving because he was ill and struggling, and I think she was very much led by him as he was the older sibling. So, she didn’t think she could move either. It was only when she was introduced to other children that she began engaging.*”

(PT1)

#### 3.8.3. Learning by Example: Caregiver Role Modelling

Children made it a priority to draw their family, and participants explained how extended relatives frequently offered extra play opportunities and additional positive role modelling:

“*That’s my mum, and my dad, and me, and my brothers* [in the drawing]. *My family. All my family. We are holding hands and going to the park.*”

(CH8)

“*We have cousins come over, and on Saturday we were going to the zip wire park. So, it was ‘go phone your cousin* [to older child] *and see if he wants to come.’ Next minute we are all off to the park together and it gets them moving a little bit.*”

(TA1)

“*My brother-in-law… The kids were round his house the other day. He got some boxing gloves out and played with the kids in his home boxing gym. It’s more fun sometimes when it’s with others rather than at home. They see it as a fun activity going round to their uncle’s house.*”

(PT10)

But it was parents who emerged as the most influential facilitators of children’s play, which children consistently described as more enjoyable when parents joined in:

“*I make obstacle courses… When I do a grown-up obstacle course, mummy and daddy need to jump over the sofa. I am going to challenge mummy, and if she doesn’t do it, she has to sit in that chair.*”

(CH2)

“*More fun* [with mum] *… Because we are bouncing together.*”

(CH3)

Participants believed that when parents joined in, children enjoyed play more and felt more confident and creative with this support:

“*It’s important to me that I join in and play with the kids. It’s part of forming those memories together. Thinking back to when they were really little it was riding on daddy’s back and I am pretending to be a donkey or whatever for them, running around and swinging them.*”

(PT9)

“*It makes him secure, and he is happy if his parents are showing an interest and joining in with what he’s doing. The child does benefit from that and grows up feeling supported.*”

(GP3)

“*I think children play for longer if parents are involved. You can push their imagination. When the children are getting bored in the garden and I’m out there, we might set up different zones and I’ll be like, ‘let’s go into the mud kitchen’, or ‘let’s have a go at swing ball in that zone.’*”

(TA1)

Importantly, participants felt that parents not only enhanced play and wellbeing, but also acted as role models for PA and FMS, with children closely observing and imitating their behaviours:

“*I’ve got an exercise mat and a fold-up exercise bike. When I do it my daughter watches and she tries to join in as well. I’ll get my water bottle and put it on the chair, and then she will get her water bottle, and she’s literally copying exactly what I’m doing. She will definitely recognise when I am doing these physical activities and try and copy.*”

(PT1)

“*Parents can hugely benefit children’s physical development because they are role models. It is something you can do as a family and enjoy it together. If a child can see their parents doing something like that, it is motivation isn’t it. That motivation to do well and achieve.*”

(TE2)

Furthermore, participants noted that children with more active and engaged parents tended to be more active themselves and demonstrated greater confidence and competence in their FMS:

“*You can tell the difference physically between the children whose parents are active and do take their children out compared to those who don’t. There is a difference. You can see it.*”

(NW1)

“*I think definitely when I look around peer groups and friends at school and stuff, the more active parents tend to have the more active and confident children. I think it is probably what you have been brought up with.*”

(PT8)

Whereas when parents were less engaged, participants felt that children struggled with play, had poorer FMS, and sometimes misinterpreted normal exertion as discomfort or worry:

“*I think lots of the children want to go out and play, but they don’t have those role models to teach them how to play or use equipment safely. Parents are not always engaging.*”

(TA1)

“*If you don’t have parents that are physical, then that becomes the norm. If they are not modelling those behaviours, then how can we expect the child to suddenly do it? … Some children won’t run because they get out of breath quickly. Well, it’s normal to get sweaty. That child is not receiving the right messaging that this is normal and fine.*”

(PT3)

“*I am just sitting still. I get unbreathed when I run…it means I get tired out sometimes. Sometimes it makes me angry.*”

(CH4)

#### 3.8.4. Intergenerational Relationship Dynamics

Participants noted that, due to parents working, it was often grandparents who provided close, valued involvement in children’s daily routines and care:

“*Grandparents, because of the cost of living, careers, women having children later in life, those grandparents are becoming the primary carers. Grandparents are part of that extended family support network.*”

(FP2)

“*Grandparents are very involved in their children’s lives now. My mum had my daughter from when she was 18 months when I went back to work, and she really took to my mum. It was a really positive experience. It’s a different person, in a different place, in a different way.*”

(TE3)

Participants highlighted how grandparents added fun and novelty to childcare, encouraging children to be active and sometimes supporting the development of specific FMS:

“*I have drawn cars and me and my nanny. This is my house and this is nanny’s house. That is us sleeping in her bed…* [asked why they had drawn nanny’s house] *…Because it’s different, and she is very nice, and very silly.*”

(CH4)

“*Her grandad helps with childcare. She never stops when she goes there. It’s constant and she comes home knackered. She enjoys being there with him.*”

(PT6)

“*I had one child where we were doing a little bit of cricket, and I realised he had really good skills. It transpired that his grandad was a cricketer, and he had been playing cricket with his grandad. His mum was so pleased that we had noticed. That was brilliant.*”

(TE2)

This was not always the case, however, as participant reports highlighted that grandparents’ abilities and attitudes towards PA varied considerably:

“*My father-in-law, who is 78, competes on the track and does park runs, and my children watch him. So, it’s having those people around you setting the example. But we went on holiday with my dad and he couldn’t walk far before he had to sit down. So, he sat in the apartment and watched TV all day, and my girls were constantly asking me questions about this behaviour.*”

(PT3)

“*I think it varies with grandparents. My side of the family aren’t very active, so if we go to my mum’s house, we don’t move from the sofa in the living room. But if we go to my partner’s family, it’s a lot more active and we are out in the garden running and climbing.*”

(TA1)

Some participants expressed doubt about grandparents’ ability to support active play and FMS, and also worried about overwhelming them with additional caregiving duties:

“*My mum isn’t very mobile, and she can’t physically get up and down well. My children could have stayed with her in the day, but we worried they might not get much out of it, so we chose to send them to nursery instead.*”

(TA1)

“*They would be restricted playing anything that involves getting up and down or running due to their age and fitness. They would struggle at a soft play centre, whereas I can do all that. I think older grandparents do have a lot to offer childcare wise, but less so for physical activity.*”

(PT5)

“*We rely a lot on grandparents at home looking after them and I think it’s hard enough for them just trying to keep their head above water doing the school run and giving them their tea without us adding anything else to their responsibilities.*”

(PT1)

Grandparents acknowledged their limitations, but often adapted activities to ensure continued participation, or simply provided motivation and encouragement when this was not possible:

“*I will get down on the floor and play with her, then she will say, ‘come on grandad we are going for a walk.’ She sees that I will be struggling back to my feet, but she understands and respects that. Grandma might make a joke and say, ‘grandad is as slow as a snail.’ So, if anything we are effectively bringing that into their play by making it a joke or making it fun.*”

(GP1)

“*As we get older, we might lose the ability to grip and that could become a problem. But then you will find alternative tasks. So, it might be a case of bouncing the ball with them instead of trying to catch it.*”

(GP2)

“*They can still be encouraging them to climb or to go down a slide or whatever. Or in the garden. Just be with them so they aren’t by themselves. Definitely from the motivational side of things, grandparents would have a part to play that way. Just not joining in as much.*”

(PS1)

One practitioner called for additional guidance for those grandparents who are physically limited on how to support children’s play activities and FMS development:

“*We have grandparents who are very fit and active, but then those who have got limitations and health needs and may not be able to join in with play. So, information must not only go out to parents but also to grandparents so they can either join in or learn how to support them.*”

(FP2)

#### 3.8.5. Children’s Personalities and Bidirectional Relationships

Participants discussed how children’s personalities influenced PA and FMS engagement, with outgoing children more likely to join in and persist, and reserved children being reluctant to participate:

“*I have two children who are very much opposite. My oldest is very reluctant, isn’t really interested, not as confident. My youngest wants to be the best and will keep trying until she is the best. She is very driven and determined. If we were at the park and kids were playing football, she would be the one that would go and join in.*”

(PT1)

“*We had a child in a session that was very introverted, but also a child that was the polar opposite. We did this speed bounce competition, and the extroverted one was bouncing, bouncing, bouncing, determined to beat the score. While the shy one jumped a couple of things, then just walked off. So, you could tell how their personalities were dictating how they engaged.*”

(PS1)

Participants explained that certain personality traits influenced not only children’s willingness to demonstrate skills, but also how they were able to respond to guidance:

“*She was trying to go down the fireman’s pole, and I watched her out the window do it by herself. But the other day I told her to go down while I was standing at the bottom, and she didn’t want to. I couldn’t understand why she did it perfectly well the other day but not for me.*”

(PT3)

“*I see very diverse groups. We have the stereotypical boys who will just run and climb and have their own little gang. Whereas you have a couple of quieter children who need that extra nurturing and I am drawn to them in the playground because they need that support, and they are more accepting of me joining in their play because they are wanting that help.*”

(TA1)

“*The energetic ones are the easiest to engage. But you can also have problems with these. There’s a lack of focus. You give instructions but they are so excited they can’t take it on board. On the other side you have the reserved academic type who may not want to do the activity, but they can tell you exactly what you said. You need adaptation for these different personalities.*”

(FP2)

Participants noted the difficulty parents sometimes faced when children responded differently to them than to educators or other adults, which required parents to adapt their approach:

“*I don’t think kids always respond to parents as well as they respond to a third party, like a coach. I think they can get a bit too comfortable with their parents, so are more likely to fob you off compared to someone else. Someone who is just there to teach them that skill.*”

(PT2)

“*I think there is a difference between your parents teaching you compared to somebody else teaching you. My daughter loves school and listens really well to the teachers. But when I try to teach her, I have to do it in a slightly different way. I think it is her own personality that is a factor in trying to teach certain things as a parent and how she responds.*”

(TE3)

Several participants argued that parents often struggle to respond to children’s personalities and interests, stressing the need for perseverance, flexibility, and tailored approaches to better suit their child’s individual needs for FMS support:

“*I think on top of personality, perhaps parents don’t always persevere with it because they might think, ‘right, I can’t do this because it’s not working.’ So, they will leave it for a teacher or a third party instead of persevering or trying to do it differently.*”

(TE3)

“*I think parents should be aware of what their children like playing with or like engaging with. If they are into something then you should make the activity more about that, and I think that would be helpful.*”

(PT4)

“*Not all children are the same or like the same activities. My son absolutely hates going on swings. He doesn’t like the motion, and it scares him. So, we had to change activities and he loves to climb. But not every child is going to like climbing at the park.*”

(TA1)

Participants also emphasised letting children take the lead in activities, providing the autonomy for them to choose what they enjoy rather than forcing participation:

“*I feel that a child should do some kind of activity, but I think they should be able to do something that they chose to do because they enjoy it. For me that was football. For my friend that was dancing. For someone else that might be walking. As long as we are doing something. I think that autonomy, that choice is important.*”

(TE1)

“*Nothing should be seen as a chore. Don’t force that child to do something because they are going to think of it as a punishment. If they enjoy it, they will do it, and then they will do it again… Some parents have the attitude of, ‘I’m the adult and you’re the child.’ That doesn’t work.*”

(GP2)

“*They take the lead. You encourage. If perhaps you don’t want them to do something a certain way you could go, ‘maybe this would work better.’ You encourage them to perhaps turn it around into something else, but generally you need to go by what they want and how they want to play.*”

(PT10)

## 4. Discussion

### 4.1. Key Findings

To the best of our knowledge, this is the first study to gather and collectively explore perspectives from educators, caregivers, and 3–5-year-old children. Each group offered valuable and complementary viewpoints that enhanced the understanding of family involvement in children’s FMS development. In line with gaps in the literature, fresh insights were obtained from family hub practitioners and grandparents, as well as from 3-year-old children using a creative draw-and-tell approach. Key findings revealed a concerning breakdown in communication channels, which prevented important messages on PA and FMS from reaching families and left existing guidance essentially invisible to the public. Differences were observed between parents, who questioned grandparents’ physical ability to support children’s FMS, and grandparents, who felt they could adapt or assist indirectly. No children drew screens or electronic devices, which was an unexpected finding given how participants reported their frequent use within daily family routines. Meanwhile, participants expressed a preference for digital guidance to support motor development at home. These findings carry important implications for the future development of family-centred approaches to support PA and FMS in early childhood and for the prevention of early motor and behavioural difficulties. They also align with established pathways linking screen time, reduced movement opportunities, and broader caregiving-related factors to increased motor and behavioural risk in early childhood.

### 4.2. Messaging

Caregivers were unfamiliar with the UK CMOs’ PA guidelines for children and were not receiving relevant messages from professionals on the importance of movement, suggesting current channels for messaging are ineffective. While a lack of caregiver awareness of the UK guidance and FMS has been identified previously [48,56,57], this study provides novel insights into the structural weaknesses within current communication pathways and offers recommendations for improving how guidance is shared. The value of impactful messaging is illustrated by the contrasting actions of informed and uninformed caregivers. Research has demonstrated that when caregivers are unaware of the PA guidelines, they are less able to judge children’s PA levels [56,57]. Further, when they lack understanding of FMS, they often fail to recognise the long-term importance of skill development [17,24]. By comparison, those with a better understanding and who hold stronger beliefs tend to model skills [58] and monitor PA more effectively [59]. Given how both current participants and previous studies [17,58] placed caregivers at the heart of children’s activity behaviours, and the role of PA in driving FMS development in early childhood [23,60], it is imperative that guidance reaches them to promote greater family support of PA and FMS development in the early years.

No educators in this study could recall the UK PA guidelines for children and suggested that training inadequacies were leaving teachers with little confidence to discuss physical development with families. Similar educator uncertainty about the current UK PA recommendations [7], and about how to support and sustain these in practice [61] has been reported previously. Notably, a recent national review [62] has argued that insufficient training is contributing towards gaps in understanding, hindering educators’ ability to promote movement to families. Although not exclusively focused on the early years, evidence points to comparable limitations in UK healthcare professionals [63,64]. Considering that practitioners act as intermediaries between policy and families [65], this reflects a significant breakdown in communication pathways across multiple channels. Despite UK PA guidance being well established, dissemination and implementation is passive and appears to extend little beyond publication [66]. As such, it is perhaps unsurprising that professionals struggle to cascade information to families. This is particularly concerning, as caregivers in this study stated that, although the guidance is readily available and could be sought independently, they relied heavily on explicit signposting from practitioners and are therefore being left poorly informed and ill-equipped to support children’s PA and FMS without this guidance. Therefore, PA guidance must be actively integrated into early childhood professional development so educators can more effectively inform caregivers on the importance of movement.

Educators were more familiar with the term “gross motor skills” than with FMS. Recent survey data show that only 58% of 137 early years practitioners [67] and 15% of 853 primary school teachers [68] recognised the term FMS. This is a worryingly low level of awareness that may be undermining their ability to accurately convey FMS concepts to families. However, a recent scoping review that used nine different search terms to capture the related literature [5] provides a clear example of the considerable variation in FMS-related terminology that may contribute to unfamiliarity with the term. Research has suggested that many educators understand and support the underlying principles of motor skill development but typically rely on gross motor terminology from the EYFS [69] rather than technical FMS language [67]. Considering the wide variation in research terminology, and the fact that practitioners rely on familiar EYFS language, there is a conceivable mismatch between the two areas that may be driving ongoing terminology confusion in educators. Closer collaboration between research and practice is needed to help establish consistent vocabulary, which would enhance educators’ clarity and confidence to integrate FMS into routine discussions with caregivers.

The finding that participants were more aware of simple activity messages and themes like “10,000 steps” and “The Daily Mile” than the official PA guidelines for children presents a clear opportunity to learn from the dissemination strategies underpinning these campaigns. Their growth in prominence and influence may stem from the fact that, unlike the PA guidelines, their messaging is actively mobilised and extensively promoted by charities, public health bodies, and media channels to ensure adoption in schools, workplaces and communities [70,71]. These aspects appealed to current participants, who suggested that the UK PA guidelines for children might reach families more effectively through increased media coverage and planned social media campaigns, and by using recognised public figures as part of these interventions. In support of this, recent evidence has stated that message recall is generally low, and they are more likely to reach caregivers if campaigns are made highly visible and repeated through multi-platform approaches [72]. Furthermore, previous UK campaigns aiming to raise awareness of the PA recommendations and the benefits of movement have received sporting celebrity endorsement from Andy Murray [73] and Joe Wicks [74] in this respect. Thus, future strategies for promoting children’s PA and FMS development should include broader public-facing campaigns and mirror the communication mechanisms that have helped these branded health initiatives gain traction.

Participants suggested families could benefit from brief, easy-to-digest digital content to support children’s FMS at home, delivered through digital platforms such as YouTube or mobile applications. Participants stated that this approach would be convenient, flexible, and compatible with existing family routines and time pressures. Similar feedback was provided by caregivers in a recent home-based motor skills intervention, who called for YouTube videos instead of written guidance, which they perceived as text-heavy, prompting authors to propose pairing minimal text with video demonstrations [75]. YouTube and digital video platforms were also instrumental in the remote delivery of PE, and provided accessible content to support families’ PA during the COVID-19 pandemic [74,76], highlighting its potential both for family messaging and direct support of children’s motor learning. In comparison, mobile applications have gained momentum as an effective method for engaging families in short, home-based sessions, and have consistently improved children’s motor competence [21,77,78]. Moreover, mobile applications can play an important role in sustaining family participation by enhancing caregiver confidence, enabling them to support practice independently [21,78]. Despite these digital approaches aligning with current participant preferences, the use of YouTube for family FMS support is yet to be thoroughly evaluated, and mobile content continues to be limited by small sample sizes and uncertain fidelity. However, digital resources clearly appeal to families and may improve guidance and interventional support of FMS in the home environment. Expanded research is needed to optimise their use in supporting family engagement in children’s FMS development.

### 4.3. The Role of Grandparents in Children’s FMS Development

Participants identified grandparents as influential members of the family unit who were frequently relied upon by parents for childcare, while grandparents themselves believed their role could extend beyond childcare to play a more meaningful role in supporting children’s FMS. Findings correspond with broader evidence that has similarly reported on the increasing prominence of grandparents within contemporary family structures, often providing daily care and educational support to young children [79,80]. However, while recent Australian-based studies have begun to explore the barriers and facilitators to grandparents promoting PA to grandchildren [81,82], little attention has been given to how their involvement might also support children’s motor development. Within the UK, research has examined how grandparents contribute to a family culture of PA [83], but offers no insight into their role in shaping children’s movement competencies. The present study therefore offers a novel contribution by beginning to address this gap in the literature and highlights grandparents as a potentially important, yet underexplored, resource for supporting children’s FMS development within the home environment. Further inquiry is evidently needed to determine how grandparents’ involvement can be most effectively leveraged to support children’s FMS development.

Parents were unsure about involving grandparents in FMS support due to concerns that age or health issues might limit their ability to engage with children. Although derived from grandparents’ own perspectives rather than parents’, previous research suggests that mobility and health limitations can negatively impact children’s PA opportunities while under grandparental care [81]. Moreover, parenting is argued to be more conducive to FMS development than grandparenting, as parents are reportedly more likely to physically engage with their children [84]. While physical capability has featured prominently in explanations of grandparental PA behaviours, our data revealed grandparents’ strong sense of responsibility to keep children safe, accompanied by a fear of blame should an injury occur. This implies that grandparents may avoid PA not just because of their own physical limits, but also to prevent causing harm to children. It is also important to recognise the considerable diversity in health and functional ability in the UK’s older adult population [85], reinforcing the notion that many grandparents will remain physically active, fit and capable of providing active, high-quality childcare. Indeed, the motives for PA participation in older adults include being able to provide physically active care for their grandchildren [86]. On the balance of evidence, grandparents may be more directly involved in children’s FMS development than currently assumed. Encouragement and reinforcement from parents could help reduce perceived risks and fear of blame, supporting grandparents to engage more confidently.

Regardless of whether conservative behaviours occurred due to functional status or risk mitigation, grandparents with physical constraints felt they could adapt to remain involved in children’s activities, even if only indirectly. Grandparents involved in previous research have similarly seen themselves as facilitators and motivators rather than active participants [81], and are capable of instilling positive activity habits across generations [83]. Furthermore, grandparents have previously responded to physical limitations by integrating children’s activity into everyday routines, facilitating peer engagement, and creating supportive spaces that promote movement [81]. This highlights that grandparents with mobility and health challenges can still influence children’s FMS indirectly through motivational and behavioural support and role modelling, without needing to be physically involved. Given their growing familial influence and considerable access to grandchildren, grandparents’ involvement in FMS support could provide children with additional opportunities to practice skills and help ease time pressures on parents. To ensure this is inclusive and realistic, tailored, age-appropriate guidance may benefit those grandparents who are physically constrained. This guidance should be offered in multiple formats, including paper copies [87], as preferred by the grandparents in this study, to ensure these messages reach this group.

### 4.4. Family Screen Time Behaviours

Participants were concerned that family screen time was displacing active play and harming children’s FMS, broader development, and sleep. Yet, despite being embedded in daily family routines, no children drew screens or electronic devices, which contrasts with the more frequent appearances of screens in previous draw-and-tell investigations [36,37]. Current participants felt children’s silence on the topic may be symbolic of habitual and normalised screen use that has led children to view it as a routine part of daily life rather than a form of play. This may be conceivable given how early and often children are exposed to screens, with caregivers using devices in public to occupy children [88] and at home to manage tasks and maintain calm [89]. These behaviours are concerning in light of the well-established harms of excessive screen use, including poorer FMS [90], language delays [91], reduced cognitive and social skills [92], and disrupted sleep [93]. Considering that research has shown screen behaviours to be deeply influenced by family patterns [89,94], any attempt to address this issue must therefore target the family system as a whole, rather than concentrating on children in isolation.

Further participant concerns were raised about the lack of clear and accessible national guidance on appropriate screen time for young children. Despite ongoing societal anxiety, the stance of the UK CMOs’ commentary on screen-based activities for children was, until very recently, that the evidence was not sufficiently robust to support the publication of specific guidance on optimal amounts of screen use [95]. Although this cautious position was understandable, the sustained lack of updated guidance left caregivers uninformed about what levels or types of screen use may be harmful and why, and may have contributed to ongoing caregiver ambivalence around screen use in children. In contrast, Canada’s 24-h Movement Guidelines [96] take a more multifaceted approach, acknowledging screen time, sleep, and PA as interrelated 24-h healthy movement behaviours that can collectively influence children’s ability to achieve movement recommendations. Research has found that children who meet all three components tend to develop stronger FMS at follow-up, supporting overall child health and wellbeing [97]. As such, future iterations of the UK PA guidance should consider adopting a similar approach to the Canadian model by incorporating advice on both screen time and sleep to help caregivers support healthier screen time behaviours in children. This long-term direction is becoming increasingly likely following recent developments. Amid growing political momentum, the UK government has begun to address this issue by releasing its first dedicated guidance for parents of young children on screen time [98].

### 4.5. Evaluation of Children’s Draw-and-Tell Methodology

Recognising the importance of learning from the process of conducting research in this area and sharing learning with interested stakeholders, the draw-and-tell methodology in this study built on previous applications in early-years children [36,37] and successfully captured valuable insights from 4–5-year-old participants. Unlike these earlier studies, children completed the task in the company of an adult rather than a peer due to concerns about copying and logistical constraints. Though this may have affected children’s honesty, as they may have felt uncomfortable sharing negative experiences in front of their caregiver. Future community-based research should consider this limitation. For 3-year-olds, even with additional methodological adaptations, interviews were more challenging and contributions narrower than those of older children. One child from this group lacked the language skills to articulate meaning, leading to their withdrawal. But the remaining 3-year-olds responded positively to the play-based approach, which helped maintain their attention and produced richer data. Although it is possible that some responses may have been influenced by the play itself, at times their responses were comparable to some of the 4–5-year-olds, suggesting these adjustments may effectively support 3-year-old participation. Expanded inquiry is needed, since so few participated. Of further note, one parent proposed including caregiver input to provide additional context for children’s responses, though this could also introduce bias by shaping or diluting children’s own perspectives.

### 4.6. Practical Implications

Current communication channels for key PA messages are largely passive and ineffective, and the UK PA guidelines for children are not fully integrated into professional training, leaving both families and the intermediaries responsible for sharing this information unaware of core messages on PA and FMS. Guidance should be made clear and accessible and actively promoted, first within practitioner education, and then across early education, healthcare, and community settings. Further, it should be recognised that PA promotion occurs ‘upstream’ and before professionals get involved [99]. Grandparents play an important role in families and could provide further support of children’s FMS, but tailored guidance is needed for those whose age or health limits their participation. Multi-component guidance is also needed to address PA, positive screen use, and sleep, reflecting the prominence of screen use in modern society and families’ preference for digital FMS resources and this may evolve in future updates of the CMO guidelines. Findings from this research will be shared through relevant academic and professional networks, including Alliance 4 Children [100], and the International Motor Competence Research Consortium (I-MDRC) [101], both of which work to advance children’s early development, PA, and motor skill research. Dissemination through these networks may raise awareness of the issues and mechanisms affecting the communication of guidance, support the cascade of evidence, and inform policymakers about improvements needed to better communicate PA and FMS to families.

### 4.7. Limitations and Strengths

Despite targeted recruitment efforts, this study was limited by its predominantly White British sample, thereby restricting the generalisability of findings to the wider UK population. Future research should include translated materials and engagement with community leaders to prioritise the participation of underrepresented ethnic minority groups [102]. A further limitation arose from recruiting a diverse range of educator roles, which led to several numerically small subgroups and limited depth of insight. This may have affected the transferability of findings, and recruitment might have benefited from the application of minimum quotas per stakeholder group [103]. The final limitations relate to the complexities of working with young children, meaning these findings should be interpreted with caution. Also, too few 3-year-olds participated to fully determine whether refinements to the draw-and tell methodology were effective in this group, highlighting the need for further research. Finally, the decision to exclude children with SEND may further limit the transferability of the findings and should be considered in future work.

Limitations were balanced by notable research strengths. The study considered a diverse range of stakeholders from multiple underrepresented groups, which provided unique insights. This included the meaningful representation of male viewpoints in fathers, grandfathers, and educators. Fathers, in particular, are relatively absent from children’s research [104], and their inclusion added valuable male insights on family needs. An additional strength was that stakeholders were offered the opportunity to share their preferences for receiving information and guidance on FMS, a proactive approach rarely employed with this population, which may inform future programme and policy design. Further strengths can be found in the flexible methodological approach that facilitated important discussions with 3-year-olds, and the interview format that accommodated a wide range of participant needs, therefore improving geographical reach [50].

## 5. Conclusions

Caregivers are central to children’s FMS development, while the family home and community offer diverse opportunities and social experiences that nurture their motor skills. However, a concerning breakdown in communication pathways is preventing information on the importance of FMS from reaching families. Clear and impactful messaging and dissemination strategies are urgently needed to actively promote FMS concepts and the UK PA guidelines for young children across early education, healthcare, and community settings. Guidance should provide age-appropriate support for grandparents, who are motivated and well placed to be more meaningfully involved in children’s motor development. UK PA guidance must also evolve to include multi-component advice on sleep, PA and positive screen use, particularly given stakeholder preference for digital FMS resources. These findings may have important implications for future policy and family-centred approaches to support FMS in early childhood.

## Figures and Tables

**Table 1:** Characteristics of adult participants.

	*n* (%)
**Gender**	
Male	7 (30.4%)
Female	16 (69.6%)
**Ethnicity**	
White British	22 (95.7%)
Black British Caribbean	1 (4.3%)
**Age (Years)**	
18–34	5 (21.7%)
35–54	12 (52.2%)
55+	6 (26.1%)
**Caregiver/Job Role**	
Parent	11 (47.8%)
Grandparent	4 (17.4%)
EYFS Teacher	1 (4.3%)
EYFS Teaching Assistant	1 (4.3%)
Nursery Worker	1 (4.3%)
Primary Teacher	2 (8.7%)
Family Hub Practitioner	2 (8.7%)
PE Specialist	1 (4.3%)

EYFS: Early Years Foundation Stage; PE: Physical Education.

**Table 2:** Characteristics of child participants, activity preferences, and exemplar quotes.

ID	Age	Sex	Activity	Why	Activity Type
CH1	5	F	Swimming	“*I like swimming…I like going to other places.*”	Active
CH2	5	F	Trampoline	“Because ***** [little sister] *chases me. She shares everything with me.*”	Active
CH3	3	F	Swimming	“*Because it’s nice.*”	Active
CH4	5	M	Toy Cars	“*I like to pretend*”	Sedentary
CH5	5	M	Football on the trampoline	“*I like it because it is fun. I am doing exercising.*”	Active
CH6	5	F	Dolls	“*Because I can relax a bit more.*”	Sedentary
CH7	5	F	Football	“*I don’t know. I just like it.*”	Active
CH8	5	F	Playing dress-up	“*I like to play dress-up all the time because it is fun.*”	Sedentary
CH9	3	M	Dinosaur figures	“*Because I like it.*”	Sedentary
CH10	4	M	Dinosaur figures	“*I like dinosaurs.*”	Sedentary
CH11	5	F	Hide-and-seek tag	“*Because it’s fun. Because you get to hide anywhere.*”	Active

***** indicates where a name has been anonymised.

## Data Availability

The data presented in this study are available on request from the corresponding author. The data are not publicly available due to ethical and GDPR reasons.

## Abbreviations

The following abbreviations are used in this manuscript:

CH	Child
CMOs’	Chief Medical Officers’
COREQ	Consolidated Criteria for Reporting Qualitative Research checklist
EYFS	Early Years Foundation Stage
FMS	Fundamental movement skills
FP	Family hub practitioner
GP	Grandparent
NW	Nursery worker
PA	Physical activity
PE	Physical Education
PT	Parent
SEND	special educational needs and disabilities
TA	Teaching assistant
TE	Teacher
UK	United Kingdom

## Appendix A

**Table A1:** Consolidated criteria for reporting qualitative studies (COREQ): 32-item checklist [40].

Number	Item	Guide Questions/Description	Manuscript Section Reference
**Domain 1: Research team and reflexivity**			
Personal Characteristics			
1.	Interviewer/facilitator	Which author/s conducted the interview or focus group	2.6
2.	Credentials	What were the researcher’s credentials? e.g., PhD, MD	2.2
3.	Occupation	What was their occupation at the time of the study?	2.2
4.	Gender	Was the researcher male or female?	2.2
5.	Experience and training	What experience or training did the researcher have?	2.2
Relationship with participants			
6.	Relationship established	Was a relationship established prior to study commencement?	2.6
7.	Participant knowledge of the interviewer	What did the participants know about the researcher? e.g., personal goals, reasons for doing the research	2.6
8.	Interviewer characteristics	What characteristics were reported about the interviewer/researcher? e.g., bias, reasons and interests in the research topic	2.6
**Domain 2: study design**			
Theoretical framework			
9.	Methodological orientation and theory	What methodological orientation was stated to underpin the study? e.g., grounded theory, discourse analysis, ethnography, phenomenology, content analysis	2.1
Participant selection			
10.	Sampling	How were participants selected? e.g., purposive, convenience, consecutive, snowball	2.3
11.	Method of approach	How were participants approached? e.g., face-to-face, telephone, mail, email	2.3
12.	Sample size	How many participants were in the study?	2.4
13.	Non-participation	How many people refused to participate or dropped out? Reasons?	2.4
Setting			
14.	Setting of data collection	Where was the data collected? e.g., home, clinic, workplace	2.6
15.	Presence of non-participants	Was anyone else present besides the participants and researchers?	2.6
16.	Description of sample	What are the important characteristics of the sample? e.g., demographic data, date	2.4
Data collection			
17.	Interview guide	Were questions, prompts, guides provided by the authors? Was it pilot tested?	2.5
18.	Repeat interviews	Were repeat interviews carried out? If yes, how many?	2.6
19.	Audio/visual recording	Did the research use audio or visual recording to collect the data?	2.6
20.	Field notes	Were field notes made during and/or after the interview or focus group?	2.6
21.	Duration	What was the duration of the interviews or focus group?	2.6
22.	Data saturation	Was data saturation discussed?	2.6
23.	Transcripts returned	Were transcripts returned to participants for comment and/or correction?	2.6
**Domain 3: analysis and findings**			
Data analysis			
24.	Number of data coders	How many data coders coded the data?	2.7
25.	Description of the coding tree	Did authors provide a description of the coding tree?	App D
26.	Derivation of themes	Were themes identified in advance or derived from the data?	2.7
27.	Software	What software, if applicable, was used to manage the data?	2.7
28.	Participant checking	Did participants provide feedback on the findings?	2.6
Reporting			
29.	Quotations presented	Were participant quotations presented to illustrate the themes/findings? Was each quotation identified? e.g., participant number	3.4–3.8
30.	Data and findings consistent	Was there consistency between the data presented and the findings?	3.4–3.8
31.	Clarity of major themes	Were major themes clearly presented in the findings?	3.3 & 3.4–3.8
32.	Clarity of minor themes	Is there a description of diverse cases or discussion of minor themes?	3.4–3.8

## Appendix B

**Table A2:** Participant area of residence and work locality according to the English Indices of Multiple Deprivation [44].

Caregivers		Educators		Children	
Residence	IMD	Work Locality	IMD	Residence	IMD
South Derbyshire	27,123	Derby	6214	South Derbyshire	27,123
South Derbyshire	27,123	Derby	1593	NE Derbyshire	19,259
Amber Valley	21,237	Derby	1593	NE Derbyshire	19,259
Coventry	20,973	Barnsley	21,061	South Derbyshire	17,869
Derby	18,140	South Derbyshire	16,059	South Derbyshire	17,869
Amber Valley	16,833	Derby	4322	Derby	3702
Amber Valley	16,833	Derby	1703	Derby	18,140
Derby	31,552	Derby	16,311	Derby	31,552
Derby	14,743			Leicester	Not disclosed
Derby	14,743			Derby	14,743
NE Derbyshire	19,259			Derby	14,743
Derby	23,308				
Derby	23,308				
Wigan	12,900				
Wigan	12,900				

IMD: Indices of Multiple Deprivation; NE: North-East. Note: IMD could not be calculated for one participant due to non-disclosure of full postal code.

## Appendix C

**Interview Schedule: Caregivers**

1. Can you tell me what the current UK government physical activity guidelines are for children under 5 years of age?
  - If yes, probe what these are?
2. Can you tell me what the current UK government physical activity guidelines are for children aged 5 to 18 years?
  - If yes, probe what these are?

*Note: Provide a brief overview of physical activity guidelines if not known.*

3. Have you heard of the term “fundamental movement skills (FMS)” before?
  - If yes, can you describe what FMS are in your own words?
  - If not, have you heard of gross motor skills before?
  - If yes, can you describe what gross motor skills are in your own words?

*Note: Provide a brief overview of FMS if not known.*

4. Do you feel children develop FMS naturally, or do they need to be taught or supported?
5. What might be the benefits of children having well developed FMS?
6. Do you feel the UK physical activity guidelines for children are effectively communicated to the public?
7. Have you ever been told about the importance of FMS and how to support them?
  - If not, how could communication be improved?
8. Whose responsibility do you think it is to promote FMS to families?
  - Probe why?
9. Where do you think FMS should be taught or supported?
  - Probe why this is the case.
10. Is physical activity important to you personally?
  - Probe why/why not?
  - Do you consider yourself to be a physically active person?
  - Do you feel you have strong FMS?
11. Is it important to you that your child is physically active?
  - Probe why/why not?
12. Is it important to you that your child has well developed FMS?
  - Probe why/why not?
13. Would you say that supporting your child’s FMS is a priority, or do other priorities or practical challenges get in the way?
  - Probe why/why not?
14. Do you ever intentionally encourage your child to take part in active play at home?
  - Probe why/why not?
15. Do you ever join in with your child’s active play, and do you feel caregiver participation can benefit children’s play and FMS development?
  - Probe why/why not?
16. Do you ever purposefully teach or support FMS during your child’s active play time?
  - Probe why/why not?
  - Probe for examples of this.
17. Do you feel caregivers need to have good knowledge and understanding of FMS to effectively support children’s FMS development?
  - Probe why/why not?
18. Do you feel caregivers’ own physical activity levels can influence their child’s physical activity participation?
  - Probe why/why not?
19. Do you feel caregivers need to have strong FMS themselves to be able to effectively support their child’s FMS development?
  - Probe why/why not?
20. Do you personally have the confidence to support your child’s FMS development?
  - Probe why/why not?
21. Do you ever take your child elsewhere in the community to take part in physical activity or for additional FMS teaching or support?
  - Probe why/why not?
22. How important is family to you, and what role does it play in your child’s daily activities and learning new skills?
23. Thinking about your lifestyle, family circumstances, and the area you live in, are there any barriers that make it difficult for you to support your child’s FMS development?
  - Probe what/why is the case?
24. Thinking about families in general, what factors might reduce children’s opportunities to be physically active and develop their FMS?
  - Probe what/why is the case?
25. Can you think of any factors or situations that have helped you to provide your child with more opportunities to be physically active and develop their FMS?
  - Probe what/why is the case?
26. Thinking about families in general, what factors might support children’s opportunities to be physically active and develop their FMS?
  - Probe what/why is the case?
27. Do you feel all families have equal access to facilities, groups, and initiatives that may support children’s physical activity and FMS development?
  - Probe what/why is the case?
28. Do you feel your child’s personality can influence their engagement with physical activity and FMS practice?
  - Probe why/why not?
29. Is there anything your child responds negatively to that might reduce their participation in physical activity and FMS practice?
  - Probe what/why this?
30. Is there anything that you might do to encourage your child to participate in physical activity and FMS practice if they are resistant?
  - Probe what/why this is?
31. If we ran a programme to support caregivers in helping children develop FMS at home, what do you think would be the best way to do this?
  - Probe what/why is the case?
32. Is there anything additional you would like to add on the topic that has not yet been discussed?

**Interview Schedule: Educators**

1. Can you tell me what the current UK government physical activity guidelines are for children under 5 years of age?
  - If yes, probe what these are?
2. Can you tell me what the current UK government physical activity guidelines are for children aged 5 to 18 years?
  - If yes, probe what these are?

*Note: Provide a brief overview of the physical activity guidelines if not known.*

3. Have you ever heard of the term “fundamental movement skills (FMS)” before?
  - If yes, can you describe what FMS are in your own words?
  - If not, have you heard of gross motor skills before?
  - Can you describe what gross motor skills are in your own words?

*Note: Provide a brief overview of FMS if not known.*

4. Do you feel children develop FMS naturally, or do they need to be taught or supported?
5. What do you think are the benefits of children having well developed FMS?
6. Do you feel the UK physical activity guidelines for children are effectively communicated to the public?
7. Have you ever been told about the importance of FMS and how to support them?
  - If not, how could communication be improved?
8. Whose responsibility do you think it is to promote FMS to families?
  - Probe why?
9. Where do you think FMS should be taught or supported?
  - Probe why this is the case?
10. Would you personally feel confident speaking with families about FMS?
  - Probe why/why not?
11. Do you feel that parents/caregivers’ own activity levels can influence children’s participation in physical activity and FMS practice?
  - Probe why/why not?
12. Do you feel it benefits children’s FMS if parents/caregivers take part in their active playtime?
  - Probe why/why not?
13. Do you feel parents/caregivers need to have good background knowledge of motor skills to effectively support children’s FMS at home?
  - Probe why/why not?
14. Do you feel parents/caregivers need to have strong FMS themselves to effectively support children’s FMS at home?
  - Probe why/why not?
15. Do you feel children’s personalities can influence how well parents/caregivers engage with their physical activity and FMS development?
  - Probe why/why not?
16. How important is family to you, and how important do you think it is that families are informed and engaged in this area?
  - Probe why/why not?
17. Thinking about children in your care who are competent in FMS, can you recall any examples of how parent/caregiver beliefs, behaviours, family circumstances, or the local environment may have positively influenced those children’s development?
  - Probe what/why this is the case?
18. Are there any other family-related factors or wider influences that you feel may positively contribute to children’s FMS development?
  - Probe what/why this is the case?
19. In your own opinion, what does positive family involvement in children’s physical activity look like, and how might this benefit their FMS development?
20. Thinking about children in your care who are less competent in FMS, can you recall any examples of how parent/caregiver beliefs, behaviours, family circumstances, or the local environment may have negatively influenced those children’s development?
  - Probe what/why this is the case.
21. Are there any other family-related factors or wider influences that you feel may have restricted those children’s FMS development?
  - Probe what/why this is the case?
22. In your own opinion, what are the main barriers that are preventing family involvement with FMS and how might these restrict children’s development?
23. In your own opinion, what might be the most effective way to provide knowledge and guidance to families to encourage their support of children’s FMS at home?
  - Probe what/why this is the case?

Is there anything additional you would like to add to the topic that has not yet been discussed?

**Interview Schedule: Children**

Please draw a picture of yourself playing your favourite thing at home.

1. What is your favourite thing to play?
2. Why do you like this?

*Note: Redirect child towards examples of active games like running and jumping if a sedentary activity has been drawn.*

3. Where do you play this?
  - Why?
4. Can you play this inside and outside?
  - Why/why not?
5. Does anything stop you from playing this?
  - What/why?
6. Do you ever go somewhere else to play this?
  - Where/why/why not?
7. Do you like playing this with other people?
  - Who/why/why not?
8. Would anything make this even more fun?
  - What/why?
9. Is there anything else you want to tell me about your playtime?

## Appendix D

**Table A3:** Coding tree summary of themes, subthemes, and example codes derived from thematic analysis of family FMS participation.

Theme	Subtheme	Example Codes
Messaging	Knowledge gaps in PA and FMS guidance	Not known, uncertainty, limited understanding
	Breakdown in communication channels	Terminology misalignment, lack of promotion, deficit framing
	Ownership of family education	Caregiver responsibility, educational support, shared accountability
	Family preferences for messaging and support	Digital delivery, concise resources, feasibly and accessibility
Responsibility and capability	Negotiating responsibility for FMS instruction	Division on responsibility, responsibility deflection, misplaced trust
	Capability to support FMS: competence and confidence	Perceived competence, physical limitations, perceived confidence
	Role of the caregiver: instruction vs. playful engagement	Play-based support, situational teaching, balanced approach
Rules and competing demands	Risk aversion	Injury prevention, fear of blame, societal risk control
	Caregiver-mediator limitations on outdoor activity	Weather averse, inappropriate clothing, restraining children
	Household rules	Rules preventing injury, rules preventing breakages, negative perceptions of indoor play
	Workload and time pressures	Work-family conflict, post-work fatigue, grandparental time advantage
	Academic pressures	Caregiver prioritisation of academia, cultural emphasis on academia, school-driven academic pressure
Lived environments and life circumstances	Outdoor spaces and active play environments	Active family gardens, valued community play spaces, extracurricular movement opportunities
	Structural inequalities and physical access	Gradual erosion of community amenities, geographical access, road infrastructure
	Social influences and family circumstances	Neighbourhood safety, economic constraints, low-cost solutions
	Family screentime behaviours	Screen-based regulation, lack of screen time guidance, technology-enabled activity
Relationships	Family ethos: foundations of engagement	Family importance, Parental gatekeeping, family bonding through activity
	Social interactions: peer and sibling influences	Peer socialisation, positive sibling influence, sibling conflict
	Learning by example: caregiver role modelling	Caregiver enhancement of play, imitative learning, caregiver modelling
	Intergenerational relationship dynamics	Grandparental influence, physical capability concerns, grandparent adaptability
	Children’s personalities and bidirectional relationships	Personality influence on engagement, caregiver adaption to personality, child autonomy
